# On the road to Mandalay: contribution to the Microhyla Tschudi, 1838 (Amphibia: Anura: Microhylidae) fauna of Myanmar with description of two new species

**DOI:** 10.24272/j.issn.2095-8137.2019.044

**Published:** 2019-07-18

**Authors:** Nikolay A Poyarkov, Vladislav A. Gorin, Than Zaw, Valentina D. Kretova, Svetlana S. Gogoleva, Parinya Pawangkhanant, Jing Che

**Affiliations:** 1 Department of Vertebrate Zoology, Biological Faculty, Lomonosov Moscow State University, Moscow 119234, Russia; 2 Joint Russian-Vietnamese Tropical Research and Technological Center, Nghia Do, Cau Giay, Hanoi, Vietnam; 3 Zoology Department, Mohnyin Degree College, Mohnyin, Kachin State 1111, Myanmar; 4 A.N. Severtsov Institute of Ecology and Evolution, Russian Academy of Sciences, Moscow 119071, Russia; 5 Zoological Museum of the Lomonosov Moscow State University, Moscow 125009, Russia; 6 Bansomdejchaopraya Rajabhat University, Thon Buri, Bangkok 10600, Thailand; 7 State Key Laboratory of Genetic Resources and Evolution, Kunming Institute of Zoology, Chinese Academy of Sciences, Kunming Yunnan 650223, China; 8 Southeast Asia Biodiversity Research Institute, Chinese Academy of Sciences, Yezin Nay Pyi Taw 05282, Myanmar

**Keywords:** Narrow-mouth frogs, Burma, Indochina, Magway, Kachin, Biodiversity, Taxonomy, mtDNA, Morphology, Acoustics, Advertisement call

## Abstract

We present a morphological and molecular assessment of the *Microhyla* fauna of Myanmar based on new collections from central (Magway Division) and northern (Kachin State) parts of the country. In total, six species of *Microhyla* are documented, including *M. berdmorei*, *M. heymonsi*, *M. butleri*, *M. mukhlesuri* and two new species described from the semi-arid savanna-like plains of the middle part of the Irrawaddy (Ayeyarwady) River Valley. We used a 2 481 bp long 12S rRNA–16S rRNA fragment of mtDNA to hypothesize genealogical relationships within *Microhyla*. We applied an integrative taxonomic approach combining molecular, morphological, and acoustic lines of evidence to evaluate the taxonomic status of Myanmar *Microhyla*. We demonstrated that the newly discovered populations of *Microhyla* sp. from the Magway Division represent two yet undescribed species. These two new sympatric species are assigned to the *M. achatina* species group, with both adapted to the seasonally dry environments of the Irrawaddy Valley. *Microhyla fodiens* **sp. nov.** is a stout-bodied species with a remarkably enlarged shovel-like outer metatarsal tubercle used for burrowing and is highly divergent from other known congeners (*P*-distance≥8.8%). *Microhyla irrawaddy* **sp. nov.** is a small-bodied slender frog reconstructed as a sister species to *M. kodial* from southern India (*P*-distance=5.3%); however, it clearly differs from the latter both in external morphology and advertisement call parameters. *Microhyla mukhlesuri* is reported from Myanmar for the first time. We further discuss the morphological diagnostics and biogeography of *Microhyla* species recorded in Myanmar.

## INTRODUCTION

Narrow-mouth or pygmy frogs of the genus *Microhyla* Tschudi, 1838 represent the largest genus of the Asian subfamily Microhylinae. The genus currently includes 46 species of mostly small to miniature ground-dwelling frogs (Frost, 2019). *Microhyla* frogs occur in various habitats across the East (southern China, including Taiwan and Hainan islands, and Ryukyu Archipelago of Japan), Southeast (Myanmar and Indochina, Malayan Peninsula, Sumatra, Java, Bali, Borneo, and some Philippine islands), and South Asia (Bangladesh, Nepal, Indian subcontinent to northern Pakistan in the west and Sri Lanka in the south) (Frost, 2019; Parker, 1934). Many *Microhyla* species are miniaturized, representing possibly the smallest known Asian tetrapods (Das & Haas, 2010). Taxonomic diversity of *Microhyla* is undoubtedly underestimated (Matsui et al., 2011; Poyarkov et al., 2014), with over half of currently recognized species being described within the last 15 years (Frost, 2019). Molecular phylogenetic methods have proven to be useful for uncovering cryptic diversity in *Microhyla* frogs (e.g., Hasan et al., 2014; Matsui et al., 2011, 2013; Seshadri et al., 2016; Vineeth et al., 2018; Wijayathilaka et al., 2016; Yuan et al., 2016; Zhang et al., 2018).

The Republic of the Union of Myanmar, formerly known as Burma, is the largest country of mainland Southeast Asia. However, it remains one of the least herpetologically studied areas in the region (Grismer et al., 2018a; Mulcahy et al., 2018; Zaw et al., 2019). To date, only five species of *Microhyla* have been recorded from the country and data on their distribution and variation are scarce (see Mulcahy et al., 2018; Wogan et al., 2008). Herpetological exploration of Myanmar (Burma) started with the expeditions of William Theobald in 1855 to 1873, with the first published data on Burmese *Microhyla* appearing in Mason (1860). Later, based on Theobalds’ collections from Pegu in southern Burma (now Bago, Bago Division), Blyth (1856) described a new species named *Engystoma berdmorei* Blyth, 1856, now regarded as *Microhyla berdmorei* (Blyth, 1856). This species was later found to be widely distributed across the country, recorded in Karin Biapo (Kayah State; Bourret, 1942), Chin Hills (Chin State; Shreve, 1940), and later in the Rakhine and Shan States, and Magway, Sagaing, Tanintharyi, and Yangon Divisions (Mulcahy et al., 2018; Wogan et al., 2008).

*Microhyla butleri* Boulenger, 1900, which was originally described from the Malayan Peninsula, was reported from Burma by Parker (1934, p. 131), though without voucher information. This species was later reported from eastern and southern parts of the country, including the He-Ho Valley in southern Shan State (Bourret, 1942), Kayah State (Hallermann et al., 2002; based on historical collection from an expedition of L. Fea to Burma in 1885), Yangon (Wogan et al., 2008), and Tanintharyi divisions (Mulcahy et al., 2018).

In his monograph on Microhylidae, Parker (1934, p. 140) reported on *M. ornata* (Duméril et Bibron, 1841) from Moulmein (now Mawlamyine, Mon State) and Pegu based on the collections of Theobald and from Thayetmyo (now Thayet, Magway Division) based on the collections of Watson. The species was first mentioned for the country by Mason (1860) as *Engystoma carnaticum* Jerdon, 1854 “1853”. Recent molecular studies demonstrated that *M. ornata*, previously considered to be a widely-distributed taxon, in fact represents a polyphyletic group of morphologically similar, but phylogenetically distant cryptic species (Garg et al., 2018a; Hasan et al., 2012, 2014, 2015; Howlader et al., 2015; Matsui et al., 2005; Yuan et al., 2016). These studies, however, did not include samples from Myanmar in their analyses. The recent review of Myanmar herpetodiversity by Mulcahy et al. (2018) mentioned *M. fissipes* Boulenger, 1884 (type locality “Formosa”, Taiwan) for Yangon, Sagaing, Bago, Mandalay, and Magway. In contrast, based on molecular study, Yuan et al. (2016) recently demonstrated that *M. fissipes* sensu stricto only occurs north of the Red River Valley, with populations from Indochina assigned to *M. mukhlesuri* Hasan, Islam, Kuramoto, Kurabayashi et Sumida, 2014, which was recently described from eastern Bangladesh. Hence, the taxonomic status of Myanmar populations previously regarded as *M. ornata* or *M. fissipes* remains unclear and requires further clarification.

*Microhyla heymonsi* Vogt, 1911 (originally described from “Formosa”, Taiwan, China) was first recorded from Myanmar in the He-Ho Valley (Shan State) by Bourret (1942). The species was later recorded in Kayah State (Hallermann et al., 2002), Kachin State, and Tanintharyi and Yangon divisions (Wogan et al., 2008), and more recently in the Bago and Mandalay divisions (Mulcahy et al., 2018).

Finally, *M. rubra* (Jerdon, 1854), originally described from Karnataka in southern India, was first mentioned to occur in Myanmar by Parker (1934, p. 143) based on a specimen collected by W. Theobald in Moulmein (specimen number BMNH87.2.26.24). This record was later repeated by Dutta (1997). Wogan et al. (2008) reported *M. rubra* from the Magway Division as a first record for the country, and also mentioned sympatric populations of *M. berdmorei*, *M. ornata*, and an undescribed species of *Microhyla*. Recently, Mulcahy et al. (2018) examined the 16S rRNA gene sequence of a Magway specimen identified as *M. rubra* by Peloso et al. (2016), reporting that the Magway population was not conspecific with *M. rubra* from Sri Lanka and India (Mulcahy et al., 2018, p. 117), and was therefore designated as “*Microhyla* sp. B”. Mulcahy et al. (2018) also reported on a new population of *Microhyla* from Chatthin (Sagaing Division), which could not be assigned to any currently recognized species, and which they nominated as “*Microhyla* sp. A”. Thus, the taxonomic status of these populations, as well as the undescribed species from the Magway Division mentioned by Wogan et al. (2008), is understudied and requires an integrative taxonomic approach for clarification.

**Figure 1:**
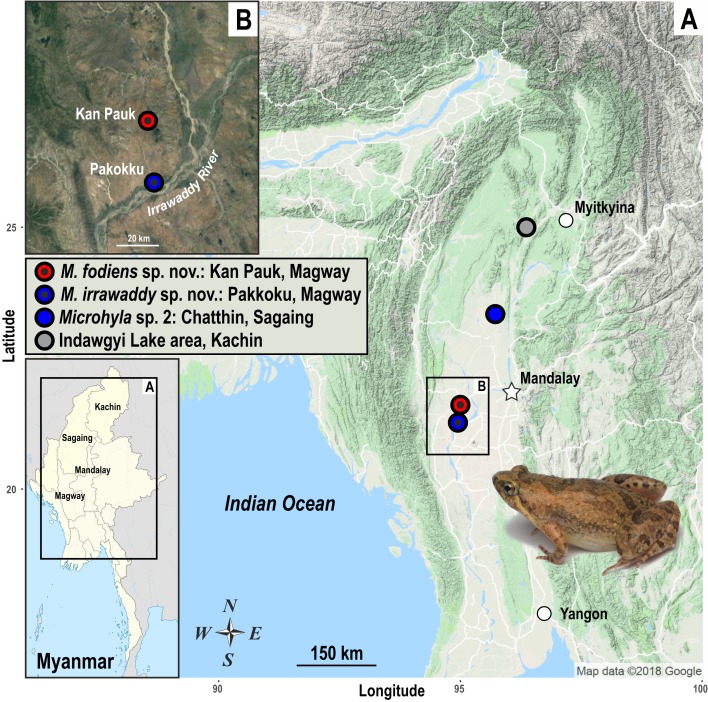
Map of Myanmar (A) showing geographic location of survey sites, including the close-up of Irrawaddy River Valley near Pakokku, Magway Division (B) Colors of localities correspond to B those used in Figure 3. Photo shows female *Microhyla irrawaddy* **sp. nov.** Photo by Nikolay A. Poyarkov. Map data – courtesy of Google Maps (2018).

**Figure 2:**
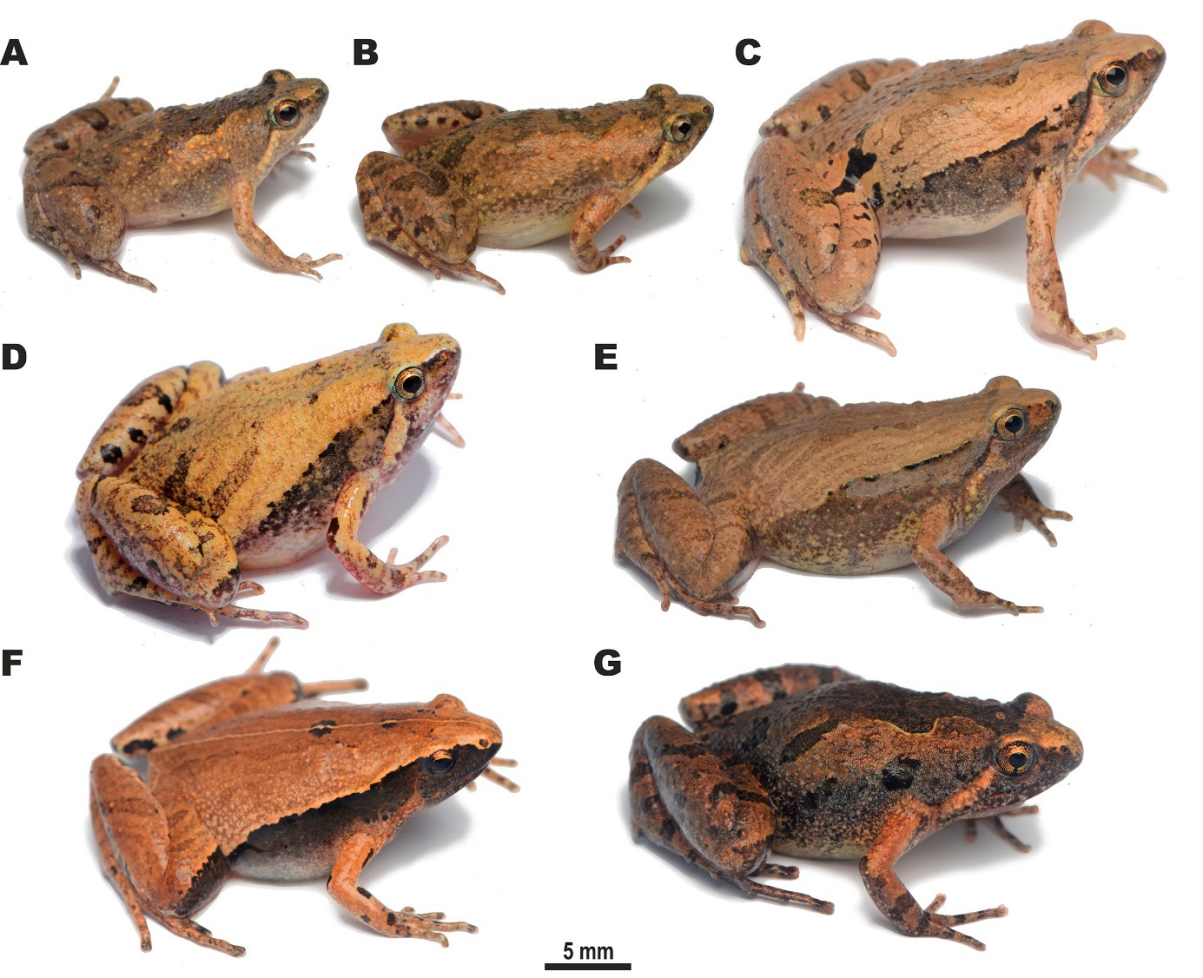
Species of *Microhyla* encountered during our herpetological surveys in the Magway Division and Kachin State of Myanmar A: Male *Microhyla irrawaddy* **sp. nov.** from Pakokku, Magway (paratype); B: Female *Microhyla irrawaddy* **sp. nov.** from Kan Pauk, Magway (paratype); C: Male *Microhyla fodiens* **sp. nov.** from Kan Pauk, Magway (holotype); D: Male *M. mukhlesuri* from Pakokku, Magway; E: Male *M. mukhlesuri* from Ingyin Taung Mt., Kachin; F: Male *M. heymonsi* from Ingyin Taung Mt., Kachin; G: Male *M. butleri* from Ingyin Taung Mt., Kachin. Photos by Nikolay A. Poyarkov.

During herpetological surveys in the Magway Division and southern Kachin State of Myanmar (see survey sites in Figure 1) in July 2018, we encountered a number of *Microhyla* specimens, which were assigned to five tentative morphospecies (Figure 2). We applied molecular, morphological, and acoustic analyses to evaluate their taxonomic status and herein describe two new species of the genus *Microhyla*.

## MATERIALS AND METHODS

### Sample collection

Fieldwork was carried out in central and northern Myanmar, including the Magway Division and Kachin State, from 14–21 July 2018. In the Magway Division, *Microhyla* specimens were collected by hand near breeding areas (e.g., temporary rain pools, paddy fields, or swamps) in two localities, including the environs of Pakokku city on the banks of the Irrawaddy River and near Kan Pauk village, Yesagyo Township, ~30 km north of Pakokku (Figure 1). In Kachin State, the *Microhyla* spp. were collected in forest clearings surrounded by montane evergreen tropical forest and bamboo forest in the Ingyin Taung Mountain, Indawgyi Lake area, Kachin State (Figure 1). Geographic coordinates and elevation were obtained using a Garmin GPSMAP 60CSx GPS receiver and recorded in datum WGS 84. Specimens were euthanized by 20% benzocaine and tissue samples for genetic analysis were taken and stored in 96% ethanol (femoral muscles) prior to preservation. Specimens were subsequently preserved in 70% ethanol and deposited in the herpetological collections of the Zoological Museum of Moscow State University (ZMMU) in Moscow, Russia, and Zoological Institute, Russian Academy of Sciences in St. Petersburg (ZISP), Russia. Other museum abbreviations include the Natural History Museum (BMNH), London, United Kingdom. In total 13 specimens of five putative morphospecies were subjected to molecular analyses (see Table 1 for details). For the two new species described below, we measured eight males, six females, and five subadult specimens (see species description sections for details).

**Table 1:** Sequences and voucher specimens of *Microhyla* and outgroup taxa used in this study

**No.**	**Specimen ID**	**Species**	**Locality**	**GenBank accession No.**	
				**12S rRNA**	**16S rRNA**
	**Ingroup**				
1	MZB Amp 16402	*Microhyla achatina*	Ungaran, Java, Indonesia	AB634598	AB634656
2	MDK 24	*Microhyla achatina*	Gede Pangrango, Java, Indonesia	AB634599	AB634657
3	KUHE 53373	*Microhyla annectens*	Genting, Selangor, Malaysia	AB634600	AB634658
4	KUHE 52438	*Microhyla annectens*	Cameron, Pahang, Malaysia	AB634601	AB634659
5	ITBC2-4360	*Microhyla aurantiventris*	Kon Ka Kinh N.P., Gia Lai, Vietnam	MH286426	
6	ITBC2-4361	*Microhyla aurantiventris*	Kon Ka Kinh N.P., Gia Lai, Vietnam	MH286427	
7	CIBBL002	*Microhyla beilunensis*	Beilun, Ningbo, Zhejiang, China	MH234521	MH234535
8	CIBBL003	*Microhyla beilunensis*	Beilun, Ningbo, Zhejiang, China	MH234522	MH234536
9	KUHE 52034	*Microhyla berdmorei*	Gombak, Selangor, Malaysia	AB598314	AB598338
10	MZB Amp 16413	*Microhyla berdmorei*	Bengkulu, Sumatra, Indonesia	AB634602	AB634660
11	MZB Amp 15270	*Microhyla berdmorei*	Paramasan, Kalimantan, Indonesia	AB634603	AB634661
12	KUHE 52373	*Microhyla berdmorei*	Besut, Terengganu, Malaysia	AB634604	AB634662
13	KUHE 21992	*Microhyla berdmorei*	Mae Yom, Phrae, Thailand	AB634609	AB634667
14	KUHE 53165	*Microhyla borneensis*	Serapi, Sarawak, Malaysia	AB598305	AB598329
15	KUHE 53938	*Microhyla borneensis*	Serapi, Sarawak, Malaysia	AB634605	AB634663
16	KUHE 40591	*Microhyla butleri*	A Luoi, A Roang, Vietnam	AB634606	AB634664
17	KUHE 44203	*Microhyla butleri*	Tainan, Taiwan, China	AB634607	AB634665
18	ZMMU NAP-08282	*Microhyla butleri*	Ingyin Taung Mt., Kachin, Myanmar	MK208937*	
19	ZMMU NAP-08283	*Microhyla butleri*	Ingyin Taung Mt., Kachin, Myanmar	MK208938*	
20	USNM 586947	*Microhyla butleri*	Yangon, Myanmar	—	MG935892
21	KUHE 32943	*Microhyla fissipes*	Huangshan, Anhui, China	AB201174	AB201185
22	CAS 215851	*Microhyla fodiens * **sp. nov.**	Kan Pauk, Magway, Myanmar	—	KM509166
23	ZMMU A5960	*Microhyla fodiens * **sp. nov.**	Kan Pauk, Magway, Myanmar	MK208926*	
24	ZMMU A5961	*Microhyla fodiens * **sp. nov.**	Kan Pauk, Magway, Myanmar	MK208927*	
25	MZB Amp 15291	*Microhyla gadjahmadai*	Lampung, Sumatra, Indonesia	AB634622	AB634680
26	MZB Amp 16328	*Microhyla gadjahmadai*	Bengkulu, Sumatra, Indonesia	AB634623	AB634681
27	KUHE 23856	*Microhyla heymonsi*	Ranong, Thailand	AB598312	AB598336
28	KUHE UN (K1845)	*Microhyla heymonsi*	Kanchanaburi, Thailand	AB201179	AB201190
29	ZMMU NAP-08277	*Microhyla heymonsi*	Ingyin Taung Mt., Kachin, Myanmar	MK208932*	
30	USNM 587130	*Microhyla heymonsi*	Bago, Myanmar	—	MG935907
	**Ingroup**				
31	USNM 587138	*Microhyla heymonsi*	Mandalay, Myanmar	—	MG935906
32	ZMMU A5966	*Microhyla irrawaddy * **sp. nov.**	Pakkoku, Magway, Myanmar	MK208928*	
33	ZMMU A5967	*Microhyla irrawaddy * **sp. nov.**	Pakkoku, Magway, Myanmar	MK208929*	
34	ZMMU A5975	*Microhyla irrawaddy * **sp. nov.**	Kan Pauk, Magway, Myanmar	MK208930*	
35	ZMMU A5976	*Microhyla irrawaddy * **sp. nov.**	Kan Pauk, Magway, Myanmar	MK208931*	
36	NCBS-AY587	*Microhyla kodial*	Mangaluru, Karnataka, India	—	MF919453
37	NCBS-AY588	*Microhyla kodial*	Mangaluru, Karnataka, India	—	MF919454
38	BNHS 5965	*Microhyla laterite*	Manipal, Karnataka, Udupi, India	KT600670	KT600663
39	BNHS 5967	*Microhyla laterite*	Manipal, Karnataka, Udupi, India	KT600671	KT600664
40	KUHE 53018	*Microhyla malang*	Serapi, Sarawak, Malaysia	AB598295	AB598319
41	BORNEENSIS 9211	*Microhyla malang*	Tawau, Sabah, Malaysia	AB598301	AB598325
42	MZB Amp 16364	*Microhyla malang*	Balikpapan, Kalimantan, Indonesia	AB634619	AB634677
43	KUHE 52556	*Microhyla mantheyi*	Temerloh, Pahang, Malaysia	AB598310	AB598334
44	KUHE 15726	*Microhyla mantheyi*	Gombak, Selangor, Malaysia	AB598309	AB598333
45	KUHE 32455	*Microhyla marmorata*	Xamneua, Houapan, Laos	AB634610	AB634668
46	DZ 1468	*Microhyla mihintalei*	Anuradhapura, Sri Lanka	—	KU214861
47	DZ 1410	*Microhyla mihintalei*	Maakandura, Sri Lanka	—	KU214857
48	DZ 1445	*Microhyla mihintalei*	Mihintale, Sri Lanka	—	KU214858
49	CIB 20070248	*Microhyla mixtura*	Sichuan, China	AB634611	AB634669
50	CIBZMH2017061203	*Microhyla mixtura*	Shaanxi, Hanzhong, China	MH234528	MH234534
51	CIB20170526001	*Microhyla mixtura*	Sichuan, Hua’e Shan, China	MH234529	MH234540
52	KUHE 22064	*Microhyla mukhlesuri*	Bangkok, Thailand	AB634608	AB634666
53	ZMMU NAP-08311	*Microhyla mukhlesuri*	Ingyin Taung Mt., Kachin, Myanmar	MK208934*	
54	ZMMU NAP-08252	*Microhyla mukhlesuri*	Pakkoku, Magway, Myanmar	MK208933*	
55	USNM 587159	*Microhyla mukhlesuri*	Mandalay, Myanmar	—	MG935905
56	USNM 587110	*Microhyla mukhlesuri*	Bago, Myanmar	—	MG935902
57	USNM 587166	*Microhyla mukhlesuri*	Magway, Myanmar	—	MG935901
58	USNM 586949	*Microhyla mukhlesuri*	Yangon, Myanmar	—	MG935897
59	IABHUF5012 BdMsp77	*Microhyla mymensinghensis*	Char Nilokhia, Bangladesh	—	AB530534
60	IABHUF5012 BdMsp78	*Microhyla mymensinghensis*	Char Nilokhia, Bangladesh	—	AB530535
61	DFBGBAU Msp 306	*Microhyla mymensinghensis*	Mymensingh, Bangladesh	—	AB530536
62	DB-Hi-FROG 12005	*Microhyla nilphamariensis*	Parbatipur, Dinajpur, Bangladesh	AB201176	AB201187
63	KUHE 12840	*Microhyla okinavensis*	Amamioshima, Japan	AB201173	AB201184
64	MZB Amp 16259	*Microhyla orientalis*	Batu Karu, Bali, Indonesia	AB634621	AB634679
65	ZSIK-A9119	*Microhyla ornata*	Karnataka, India	AB201177	AB201188
66	MZB Amp 16255	*Microhyla palmipes*	Bedegul, Bali, Indonesia	AB634612	AB634670
67	MZB Amp 16323	*Microhyla palmipes*	Bengkulu, Sumatra, Indonesia	AB634613	AB634671
68	KUHE UN	*Microhyla perparva*	Balikpapan, Kalimantan, Indonesia	AB634614	AB634672
69	KUHE 53675	*Microhyla perparva*	Mulu, Sarawak, Malaysia	AB634615	AB634673
70	BORN 22412	*Microhyla petrigena*	Maliau Basin, Sabah, Malaysia	AB634616	AB634674
71	KUHE 53743	*Microhyla petrigena*	Bukit Kana, Sarawak, Malaysia	AB634617	AB634675
72	KUHE 22113	*Microhyla pulchra*	Pilok, Kanchaburi, Thailand	AB634618	AB634676
73	KUHE 35119	*Microhyla pulchra*	Phu Luan, Loei, Thailand	AB201180	AB201191
	**Ingroup**				
74	ZMMU A5006-18	*Microhyla rubra*	Bapatla, Andhra Pradesh, India	MK208935*	
75	ZMMU A5006-19	*Microhyla rubra*	Bapatla, Andhra Pradesh, India	MK208936*	
76	MRK; released (toe tip)	*Microhyla rubra*	Karnataka, India	AB201181	AB201192
77	ATREE_MISH_1	*Microhyla sholigari*	Manipal, Karnataka, Udupi, India	KT600667	KT600674
78	ATREE_MISH_2	*Microhyla sholigari*	Manipal, Karnataka, Udupi, India	KT600668	KT600675
79	KUHE 52558	*Microhyla superciliaris*	Temerloh, Pahang, Malaysia	AB634624	AB634682
80	KUHE 53371	*Microhyla superciliaris*	Kenaboi, Negeri Sembilan, Malaysia	AB634625	AB634683
81	NHM-TU-17A-0110	*Microhyla taraiensis*	Mechi, Jhapa, Jamun Khadi, Nepal	MF496241	
82	BORN 8480	*Microhyla* sp. 1	Crocker, Sabah, Malaysia	AB634620	AB634678
83	USNM 523975	*Microhyla* sp. 2	Chatthin, Sagaing, Myanmar	—	MG935884
84	USNM 537450	*Microhyla* sp. 2	Chatthin, Sagaing, Myanmar	—	MG935885
	**Outgroup**				
85	KUHE 44148	*Calluella yunnanensis*	Pet trade	AB634626	AB634684
86	KUHE 35163	*Calluella guttulata*	Pilok, Kanchanaburi, Thailand	AB634627	AB634685
87	KUHE 52463	*Calluella minuta*	Temerloh, Pahang, Malaysia	AB598316	AB598340
88	KUHE 35182	*Glyphoglossus molossus*	Barrnta, Tak, k Thailand	AB201182	AB201193
89	BORN 8478	*Chaperina fusca*	Crocker, Sabah, Malaysia	AB598318	AB598342
90	KUHE UN	*Kaloula picta*	Pet trade	AB634628	AB634686
91	KUHE 32313	*Kaloula baleata*	Sumba, Indonesia	AB634629	AB634687
92	KUHE 33139	*Kaloula borealis*	Cheju, Korea	AB634630	AB634688
93	KUHE 35178	*Kaloula mediolineata*	Barrntak, Tak, Thailand	AB634631	AB634689
94	KUHE 22206	*Kaloula pulchra*	Nong Khai, Thailand	AB634632	AB634690
95	KUHE 37252	*Kaloula taprobanica*	Sri Lanka	AB634633	AB634691
96	KUZ 21655	*Metaphrynella pollicaris*	Fraser’s Hill, Pahang, Malaysia	AB634634	AB634692
97	BORN 8191	*Metaphrynella sundana*	Crocker, Sabah, Malaysia	AB634635	AB634693
98	UKMHC 820	*Phrynella pulchra*	Hulu Trengganu, Trengganu, Malaysia	AB634636	AB634694
99	KUHE 20497	*Micryletta inornata*	Mae Yom, Phrae, Thailand	AB598317	AB598341
100	KUHE 23858	*Micryletta inornata*	Ranong, Thailand	AB634637	AB634695
101	KUHE 35937	*Micryletta steinegeri*	Yunlin, Taiwan, China	AB634638	AB634696
102	BORN 8089	*Kalophrynus heterochirus*	Crocker, Sabah, Malaysia	AB634639	AB634697
103	USNM GZ 33787	*Kalophrynus interlineatus*	Chatthin, Myanmar	AB634640	AB634698
104	KUHE 52454	*Kalophrynus palmatissimus*	Pahang, Temerloh, Malaysia	AB634641	AB634699
105	MZB Amp 15295	*Kalophrynus pleurostigma*	Sumatra, Lampung, Indonesia	AB634642	AB634700
106	KUHE 53284	*Kalophrynus* sp.	Pulai, Johol, Malaysia	AB634643	AB634701
107	KUHE 35230	*Kalophrynus stellatus*	Pet trade	AB634644	AB634702
108	KUHE 53145	*Kalophrynus subterrestris*	Tubau, Sarawak, Malaysia	AB634645	AB634703
109	KUHE 15531	*Kalophrynus yongi*	Cameron, Pahang, Malaysia	AB634646	AB634704
110	UKM HC 279	*Gastrophrynoides immaculatus*	Negeri Sembilan, Malaysia	AB634647	AB634705
	**Outgroup**				
111	KUHE 33150	*Dyscophus guineti*	Pet trade	AB634648	AB634706
112	KUHE 35001	*Dyscophus insularis*	Pet trade	AB634649	AB634707
113	KUHE 33224	*Gastrophryne olivacea*	Dimmit, Texas, USA	AB634650	AB634708
114	MZB Amp 16265	*Oreophryne monticola*	Batu Karu, Bali, Indonesia	AB634651	AB634709
115	KUHE 33277	*Phrynomantis bifasciatus*	Pet trade	AB634652	AB634710
116	KUHE 34977	*Scaphiophryne gottlebei*	Pet trade	AB634653	AB634711
117	—	*Rhacophorus schlegelii*	Hiroshima, Japan	AB202078	

For sampling localities in Myanmar see Figure 1. Sequences generated in this study are marked with an asterisk (*); En-dash (–) denotes no data available.

### Morphological description

The *Microhyla* specimens were photographed in life and after preservation. Measurements were taken using a digital caliper to the nearest 0.01 mm, subsequently rounded to 0.1 mm. We used a stereoscopic light binocular microscope when necessary. All measurements were taken on the right side of the examined specimen. Statistical analyses were performed with Statistica 6.0 (StatSoft, Inc., 2001).

Morphometric and character terminology followed Poyarkov et al. (2014, 2018), including: (1) snout-vent length (SVL; distance from tip of snout to cloaca); (2) head length (HL; distance from tip of snout to hind border of jaw angle); (3) snout length (SL; distance from anterior corner of eye to tip of snout); (4) eye length (EL; distance between anterior and posterior corners of eye); (5) nostril-eye length (N-EL; distance between anterior corner of eye and nostril center); (6) head width (HW; maximum width of head at level of mouth angles in ventral view); (7) internarial distance (IND; distance between central points of nostrils); (8) interorbital distance (IOD; shortest distance between medial edges of eyeballs in dorsal view); (9) upper eyelid width (UEW; maximum distance between medial edge of eyeball and lateral edge of upper eyelid); (10) forelimb length (FLL; length of straightened forelimb to tip of third finger); (11) lower arm and hand length (LAL; distance between elbow and tip of third finger); (12) hand length (HAL; distance between proximal end of outer palmar (metacarpal) tubercle and tip of third finger); (13) first finger length (1FL, distance between tip and distal end of inner palmar tubercle); (14) inner palmar tubercle length (IPTL; maximum distance between proximal and distal ends of inner palmar tubercle); (15) outer palmar tubercle length (OPTL; maximum diameter of outer palmar tubercle); (16) third finger disk diameter (3FDD); (17) hindlimb length (HLL; length of straightened hindlimb from groin to tip of fourth toe); (18) tibia length (TL; distance between knee and tibiotarsal articulation); (19) foot length (FL; distance between distal end of tibia and tip of fourth toe); (20) inner metatarsal tubercle length (IMTL; maximum length of inner metatarsal tubercle); (21) first toe length (1TOEL), distance between distal end of inner metatarsal tubercle and tip of first toe; (22) fourth toe disk diameter (4TDD); and (23) outer metatarsal tubercle length (OMTL; maximum length of outer metatarsal tubercle). For the holotype description, we additionally took the following measurements: (24–26) second to fourth finger lengths (2–3FL-O, 4FL-I; outer side (O) of second and third, inner side (I) of fourth, distance between tip and junction of neighboring finger); (27–30) second to fifth toe lengths (outer lengths for toes II–IV, inner length for toe V; 2–5TOEL).

Terminology for describing eye coloration in living individuals followed Glaw & Vences (1997) and toe webbing and subarticular tubercle formulas were in accordance with those of Savage (1975). The sex and maturity of the specimens were checked by minor dissections and direct observation of calling in living males prior to collection.

Diagnosis of the genus *Microhyla* and morphological characters for comparison were taken from original descriptions and taxonomic reviews of the genus, including the following works: Andersson (1942); Atmaja et al. (2019); Bain & Nguyen (2004); Blyth (1856); Boulenger (1884, 1897, 1900); Bourret (1942); Das & Haas (2010); Das et al. (2007); Duméril & Bibron (1841); Dutta & Ray (2000); Fei et al. (2010); Fernando & Siriwardhane (1996); Garg et al. (2018b); Hallowell (1861); Hasan et al. (2014); Howlader et al. (2015); Hu et al. (1966); Inger & Frogner (1979); Inger (1989); Jerdon (1854); Khatiwada et al. (2017a); Matsui (2011); Matsui et al. (2013); Nguyen et al. (2019); Parker (1928, 1934); Parker & Osman (1948); Pillai (1977); Poyarkov et al. (2014); Schenkel (1901); Seshadri et al. (2016); Smith (1923); Stejneger (1901); Taylor (1934); von Tschudi (1838); Vineeth et al. (2018); Vogt (1911); Wijayathilaka et al. (2016); and Zhang et al. (2018).

### DNA isolation, polymerase chain reaction (PCR), and sequencing

For molecular phylogenetic analyses, we extracted total genomic DNA from ethanol-preserved femoral muscle tissue using standard phenol-chloroform-proteinase K extraction with consequent isopropanol precipitation, for a final concentration of ~1 mg/mL (protocols followed Hillis et al., 1996 and Sambrook et al., 2001). We visualized the isolated total genomic DNA using agarose electrophoresis in the presence of ethidium bromide. We measured the concentration of total DNA in 1 μl using NanoDrop 2000 (Thermo Scientific), which was consequently adjusted to ~100 ng DNA/μL.

We amplified mtDNA fragments covering partial sequences of 12S rRNA and 16S rRNA mtDNA genes and a complete sequence of the tRNA^Val^ gene to obtain a 2 481 bp length continuous fragment of mtDNA. The 16S rRNA gene is widely applied in biodiversity surveys in amphibians (Vences et al., 2005a, 2005b; Vieites et al., 2009) and, together with 12S rRNA partial sequences, has been used in most recent phylogenetic studies on Microhylinae (Matsui et al., 2011; Peloso et al., 2016). These fragments are particularly useful in studies of the genus *Microhyla* (e.g., Hasan et al., 2012, 2014, 2015; Howlader et al., 2015; Khatiwada et al., 2017a; Matsui 2011; Matsui et al., 2013; Peloso et al., 2016; Wijayathilaka et al., 2016). We performed DNA amplification in 20 μL reactions using 50 ng of genomic DNA, 10 nmol of each primer, 15 nmol of each dNTP, 50 nmol of additional MgCl_2_, Taq PCR buffer (10 mmol/L Tris-HCl, pH 8.3, 50 mmol/L KCl, 1.1 mmol/L MgCl_2_, and 0.01% gelatin), and 1 U of Taq DNA polymerase. Primers used for PCR and sequencing followed Nguyen et al. (2019) and included four forward primers: Micro-1F-12stail (ACGCTAAAATGWACCCTAAAAAGT; Nguyen et al., 2019), Micro-500F-12stail (CCACTTGAACCCACGACAG CTAGRAMACAA; Nguyen et al., 2019), 12sA-L (AACTGGGA TTAGATACCCCACTAT; Palumbi et al., 1991), L-2188 (AAAGTGGGCCTAAAAGCAGCCA; Matsui et al., 2006), and four reverse primers: Micro-600R-12stail (TAGAGGAGCCTG TTCTATAATCGATTC; Nguyen et al., 2019), Micro-1200R-12stail (AGTAAAGGCGATYAAAAAATRTTTCAAAG; Nguyen et al., 2019), R-1169 (GTGGCTGCTTTTAGGCCCACT; Wilkinson et al., 2002), and 16H-1 (CTCCGGTCTGAACTCA GATCACGTAGG; Hedges, 1994). The PCR conditions included an initial denaturation step of 5 min at 94 °C and 43 cycles of denaturation for 1 min at 94 °C, primer annealing for 1 min with the TouchDown program from 65 °C to 55 °C reducing 1 °C every cycle, extension for 1 min at 72 °C, and final extension step for 5 min at 72 °C.

We loaded PCR products onto 1% agarose gels in the presence of ethidium bromide, which were then visualized using agarose electrophoresis. If distinct bands were produced, we purified the PCR products using 2 μL of a 1:4 dilution of ExoSapIT (Amersham, Buckinghamshire, UK) per 5 μL of PCR product prior to cycle sequencing. The 10 μL sequencing reaction included 2 μL of template, 2.5 μL of sequencing buffer, 0.8 μL of 10 pmol primer, 0.4 μL of BigDye Terminator v3.1 Sequencing Standard (Applied Biosystems, USA), and 4.2 μL of water. The cycle sequencing included 35 cycles of 10 s at 96 °C, 10 s at 50 °C, and 4 min at 60 °C. We purified the cycle sequencing products by ethanol precipitation. We carried out sequence data collection and visualization on an ABI 3730xl Automated Sequencer (Applied Biosystems, USA). The obtained sequences were deposited in GenBank under the accession Nos. MK208926–MK208938 (Table 1).

### Phylogenetic analyses

To hypothesize matrilineal genealogy, we used the 12S rRNA and 16S rRNA Microhylidae dataset of Matsui et al. (2011) with the addition of sequences from several recently reported Southeast Asian *Microhyla* (Hasan et al., 2014; Khatiwada et al., 2017a, 2017b; Mulcahy et al., 2018; Nguyen et al., 2019; Peloso et al., 2016; Vineeth et al., 2018; Wijayathilaka et al., 2016; Zhang et al., 2018) and our newly obtained sequences (summarized in Table 1). In total, we obtained 12S rRNA and 16S rRNA data from 117 specimens. This consisted of 84 samples from 32 species of *Microhyla* (representing almost three quarters of recognized species within the genus), 32 outgroup sequences of other microhylid representatives, and a sequence of *Rhacophorus schlegelii* (Günther) (Sano et al., 2005), which was used to root the tree.

We initially aligned nucleotide sequences using MAFFT v.6 (Katoh et al., 2002) with default parameters, and then checked and slightly adjusted them by eye using BioEdit 7.0.5.2 (Hall, 1999) and MEGA 6.0 (Tamura et al., 2013). We determined mean uncorrected genetic distances (*P*-distances) between sequences with MEGA 6.0. We used MODELTEST v.3.6 (Posada & Crandall, 1998) to estimate the optimal evolutionary models for dataset analysis. The best-fitting model was the GTR+G model of DNA evolution, as suggested by the Akaike Information Criterion (AIC) for three partitions: 12S rRNA, tRNA^Val^, and 16S rRNA.

We inferred matrilineal genealogy using maximum likelihood (ML) and Bayesian inference (BI) approaches. We conducted ML analyses using the RAxML web server (http: //embnet.vital-it.ch/raxml-bb/; Kozlov et al., 2018), which was used to search the ML trees based on the gamma model of rate heterogeneity option. We assessed nodal confidence for 12S rRNA–16S rRNA analysis by non-parametric bootstrapping (BS) with 1 000 pseudoreplicates (Felsenstein, 1985). We conducted BI in MrBayes 3.1.2 (Ronquist & Huelsenbeck, 2003); Metropolis-coupled Markov chain Monte Carlo (MCMCMC) analyses were run with one cold chain and three heated chains for twenty million generations, sampled every 2 000 generations. Five independent MCMCMC runs were performed and 1 000 trees were discarded as burn-in. We checked the convergence of the runs and that the effective sample sizes (ESS) were all above 200 by exploring the likelihood plots using TRACER v1.6 (Rambaut et al., 2014).

In both datasets, we *a priori* regarded tree nodes with BS values of 75% or greater and PP values over 0.95 to be sufficiently resolved. BS values between 75% and 50% and PP values between 0.95 and 0.90 were regarded as tendencies. Lower values indicated unresolved nodes (Huelsenbeck & Hillis, 1993).

### Acoustic analysis

Advertisement calls of *Microhyla* sp. were recorded at breeding sites on the banks of a temporary pond in the Irrawaddy River Valley in Pakokku, Pakoku District, Magway Division, Myanmar (coordinates N21.316°, E95.053°; elevation 59 m a.s.l.) on 14 July 2018 at 2327 h and at 21.5 °C using a portable digital audio recorder Zoom h5 (ZOOM Corporation, Tokyo, Japan) in stereo mode with 48 kHz sampling frequency and 16-bit precision. The temperature was measured at the calling site immediately after the audio recording with a digital thermometer KTJ TA218A Digital LCD Thermometer-Hydrometer.

Calls were analysed using Avisoft SASLab Pro software v.5.2.05 (Avisoft Bioacoustics, Germany). Before analysis, we reduced the background noise using a low-pass filter (up to 300 Hz). All temporal parameters were analysed with the standard marker cursor in the main window of Avisoft and frequencies of the maximum amplitude of calls and pulses were measured in the power spectrum. The spectrogram for analysis was created using a Hamming window, with FFT-length 1 024 points, frame 75%, and overlap 93.75%. For graphic representation of spectrograms, we lowered the sampling rate to 22.05 kHz. Figures of spectrograms were created using a Hamming window, with FFT-length 512 points, frame 75%, and overlap 87.5%. In total, we measured 50 calls from two *Microhyla* males.

We measured five temporal parameters: i.e., call duration, number of pulses per call, duration of pulses, intervals between successive pulses, and pulse period; and two power parameters: i.e., frequency of maximum amplitude (Fpeak) of calls and of pulses. Additionally, we calculated the pulse repetition rate (pulses/s) by counting the number of pulses within each call minus one and dividing that number by the call duration. Descriptive statistics were performed using STATISTICA, v.10 (StatSoft, Tulsa, OK, USA). Most numeral parameters are given as means±*SD*, except the number of pulses per call (median±interquartile range), and the minimum and maximum values are given in parentheses (min-max).

## RESULTS

### Phylogenetic analyses

**Sequences and statistics:** Final alignment of the 12S rRNA–16S rRNA fragment contained 2 481 aligned characters, with 701 conserved sites and 761 variable sites, of which 599 were parsimony-informative. The transition-transversion bias (R) was estimated at 2.52 (all data given for ingroup only). Nucleotide frequencies were 31.64% (A), 22.82% (T), 24.37% (C), and 21.17% (G).

**Figure 3:**
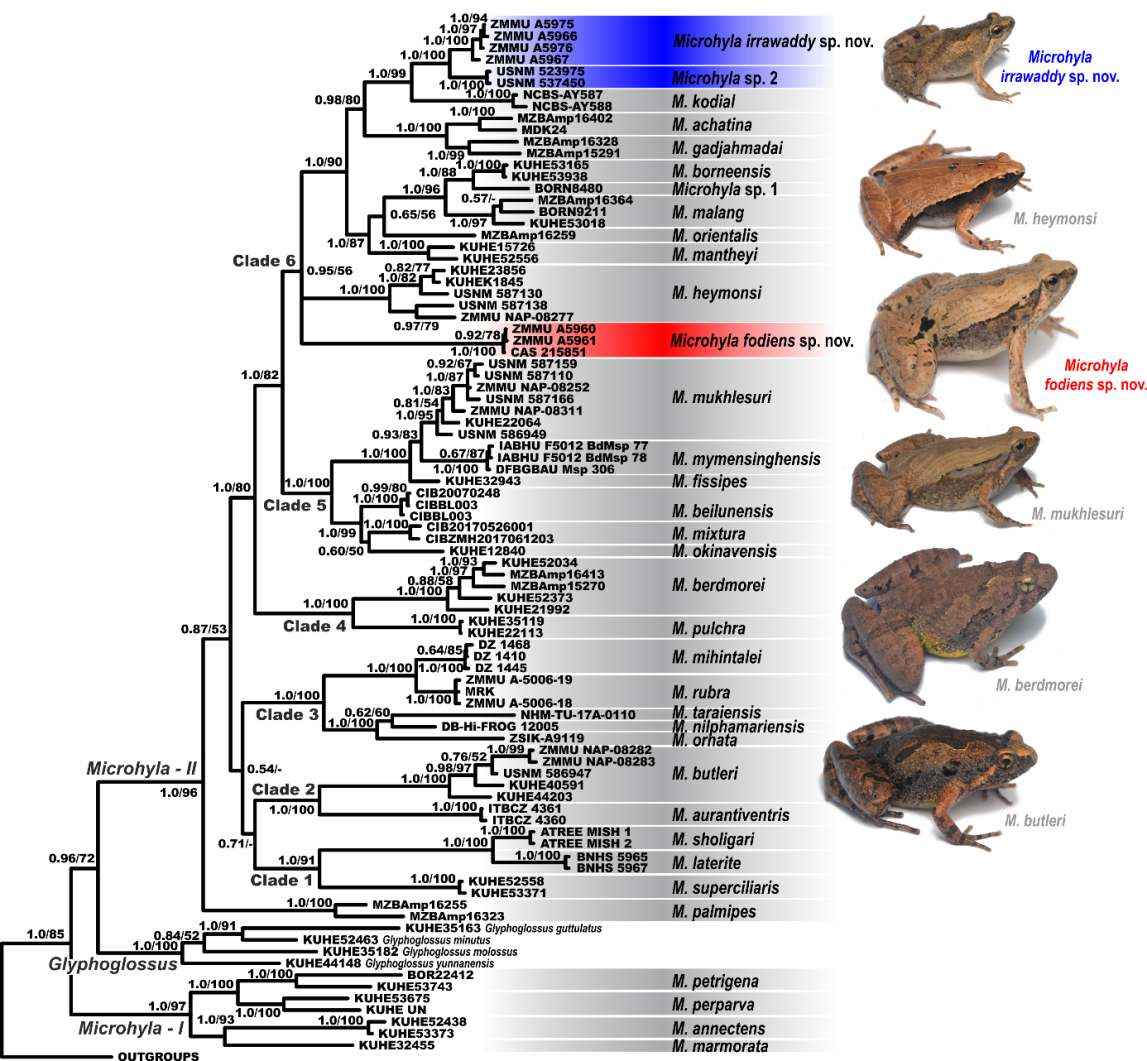
Bayesian inference tree of *Microhyla* derived from analysis of 2 481 bp long alignment of 12S rRNA, tRNA^Val^, and 16S rRNA gene fragments For voucher specimen information and GenBank accession Nos. see Table 1. Red and blue denote new species of *Microhyla* from Myanmar (see Figure 1). Numbers at tree nodes correspond to BI PP/ML BS support values, respectively. Outgroup taxa not shown. Photos showing six species of *Microhyla* recorded from Myanmar taken by Nikolay A. Poyarkov.

**mtDNA genealogy:** Both BI and ML analyses resulted in similar topologies, which differed only in several poorly supported nodes (Figure 3). The obtained topology is generally consistent with the results of Matsui et al. (2011), Peloso et al. (2016), and Nguyen et al. (2019). Analyses achieved high phylogenetic resolution at species complexes and species-level groups, with most nodes showing strong support (PP≥0.95; BS>90%). However, several major nodes showing phylogenetic relationships among outgroup taxa and major lineages of *Microhyla* presented low or insignificant levels of support.

The BI genealogy (Figure 3) inferred the following set of phylogenetic relationships:

1) Monophyly of *Microhyla* is rejected (in agreement with Matsui et al., 2011), suggesting that the genus is monophyletic with respect to *Glyphoglossus*. *Microhyla* sensu lato is thus divided into two major groups, the first corresponding to the *M. annectens* species group (*Microhyla*–I, see Figure 3) and the second encompassing all remaining species (*Microhyla*–II, see Figure 3).

2) Within the *M. annectens* species group, species are clustered into two reciprocally monophyletic clades: one joining *M. annectens* Boulenger, 1900 and *M. marmorata* Bain & Nguyen, 2004 from mainland Indochina and peninsular Malaysia (1.0/93; hereafter nodal support values given for PP/BS, respectively), and another joining Bornean species *M. petrigena* Inger & Frogner, 1979 and *M. perparva* Inger & Frogner, 1979 (1.0/100).

3) Within the second species group of *Microhyla*, *M. palmipes* Boulenger, 1897 is reconstructed as a sister species to all remaining taxa, although with low node support (0.87/53). All remaining species are grouped in six well-supported clades 1–6.

4) Clade 1 (1.0/91) joins *M. superciliaris* Parker, 1928 from the Malayan Peninsula with two species from southern India: *M. sholigari* Dutta & Ray, 2000 and *M. laterite* Seshadri, Singal, Priti, Pavikanth, Vidisha, Saurabh, Pratik & Gururaja 2016, the latter two species are closely related and form a monophyly (1.0/100).

5) Clade 2 (1.0/100) joins *M. butleri* with a closely related species *M. aurantiventris* Nguyen, Poyarkov, Nguyen, Nguyen, Tran, Gorin, Murphy & Nguyen, 2019 from the Central Plateau of Vietnam. Two specimens of *Microhyla* sp. (ZMMU NAP-08282 and NAP-08283; Figure 2G) from Ingyin Taung Mt., Kachin State, unambiguously fall into the radiation of *M. butleri*.

6) Clade 3 (1.0/100) corresponds to the *M. ornata* species group and joins a number of taxa from the Indian subcontinent and is divided in two subclades. The first subclade joins two species with stout body habitus and large outer metatarsal tubercle used for burrowing, from arid areas of southern and eastern India (*M. rubra*) and Sri Lanka (*M. mihintalei* Wijayathilaka, Garg, Senevirathne, Karunarathna, Biju & Meegaskumbura, 2016). The second subclade includes smaller species from India, Nepal, and Bangladesh: *M. ornata*, *M. taraiensis* Khatiwada, Shu, Wang, Thapa, Wang & Jiang, 2017, and *M. nilphamariensis* Howlader, Nair, Gopalan & Merilä, 2015.

7) Clade 4 (1.0/100) joins two large-bodied species, *M. berdmorei* (widely distributed across Indochina and Sundaland and also occurring in Myanmar) and *M. picta* Schenkel, 1901; the latter species has a stout body habitus and enlarged shovel-like outer metatarsal tubercle.

8) Clade 5 (1.0/100) corresponds to the *M. fissipes* species group and consists of two subclades. The first subclade joins three species of *Microhyla* occurring in East Asia: *M. mixtura* Liu & Hu, 1966 in Hu et al. (1966) and *M. beilunensis* Zhang, Fei, Ye, Wang, Wang & Jiang, 2018 from China, and *M. okinavensis* Stejneger, 1901 from the Ryukyu Archipelago in Japan. The second subclade joins taxa from the southern part of China and Indochina, including *M. fissipes* from southern China, Taiwan, and northern Vietnam, *M. mymensinghensis* Hasan, Islam, Kuramoto, Kurabayashi et Sumida, 2014 from Bangladesh, and *M. mukhlesuri* from Indochina. Two specimens of *Microhyla* sp. from Pakkoku, Magway (ZMMU NAP-08252; Figure 2D) and from Ingyin Taung Mt., Kachin State (ZMMU NAP-08311; Figure 2E) were assigned to *M. mukhlesuri* and grouped with other Myanmar specimens of this species (USNM 587110, 587159, 587166). *Microhyla mukhlesuri* and *M. mymensinghensis* form a moderately supported clade (0.93/83).

9) Clade 6 shows moderate support (0.93/56) and joins members of the *M. achatina* species group and related taxa. The stout-bodied *Microhyla* sp. (ZMMU A5960–A5961; Figure 2C) from Kan Pauk, Magway Division, are grouped and appear to be conspecific with *M.* “*rubra*” of Peloso et al. (2016) (CAS 215851) with high support (1.0/100); this species has an unresolved position within Clade 6 and is not closely related to *M. rubra* sensu stricto from India.

10) The position of *M. heymonsi* within Clade 6 is also unresolved; a specimen of *Microhyla* sp. from Ingyin Taung Mt., Kachin State (ZMMU NAP-08277; Figure 2F), is placed within the radiation of *M. heymonsi*, which is subdivided into two moderately divergent major lineages. Other specimens of *M. heymonsi* from Myanmar belong to two different lineages of *M. heymonsi* (USNM 587130 and USNM 587138).

11) A number of species from Sundaland form a subclade (1.0/87) within Clade 6: *M. mantheyi* Das, Yaakob & Sukumaran, 2007 (Malayan Peninsula) and *M. borneensis* Parker, 1928, *M. malang* Matsui, 2011, *M. orientalis* Matsui, Hamidy et Eto, 2013, and *Microhyla* sp. 1 (Borneo).

12) Sundaland species *M. achatina* Tschudi, 1838 (from Java) and *M. gadjahmadai* Atmaja, Hamidy, Arisuryanti, Matsui & Smith, 2019 (from Sumatra) form a sister lineage (0.98/80) with respect to *M. kodial* Vineeth, Radhakrishna, Godwin, Anwesha, Rajashekhar & Aravind, 2018, from southern India and two *Microhyla* sp. lineages from central Myanmar. Small-bodied slender *Microhyla* sp. specimens from the Magway Division (ZMMU A5966–A5967; A5975–A5976; Figure 2A, B) and *Microhyla* sp. 2 from the Sagaing Division (USNM 523975, 537450) form two distinct reciprocally monophyletic groups (1.0/100). *Microhyla kodial* is strongly suggested as a sister lineage to Myanmar taxa (1.0/99) (see Figure 3).

**Sequence divergence:** For uncorrected *P*-distances for the 16S rRNA gene fragment among and within the examined *Microhyla* species see Table 2. Intraspecific distances ranged from *P*=0% in a number of examined species to *P*=4.5% in *M. petrigena* (the latter may be explained by incomplete taxonomy of Bornean *Microhyla*). The interspecific distances within *Microhyla* varied from *P*=2.7% (between *M. rubra* and *M. mihintalei*) to *P*=13.1% (between *M. laterite* and stout-bodied *Microhyla* sp. from Kan Pauk, Magway) (Table 2). Genetic divergence within *M. mukhlesuri* was *P*=1.3%, within *M. butleri* was *P*=1.7%, and within *M. heymonsi* was *P*=2.3%. No genetic variation was observed between haplotypes within stout-bodied and slender-bodied species of *Microhyla* sp. from Magway (*P*=0.0%) (Table 2). Divergence between these taxa and their closest relatives was *P*=2.0% for small slender-bodied species of *Microhyla* sp. from the Magway Division if compared to *Microhyla* sp.2 from Sagaing Division, and *P*=5.3% if compared with *M. kodial*; and was *P*=8.8% for stout-bodied *Microhyla* sp. from Magway Division with *M. berdmorei* (Table 2).

**Table 2:** **Uncorrected *P*-distances (percentage) between the sequences of 12S rRNA**–**16S rRNA mtDNA fragments (below the diagonal) and intraspecific genetic *P*-distances (on the diagonal) of *Microhyla* species included in phylogenetic analyses**

**Species**	1	2	3	4	5	6	7	8	9	10	11	12	13	14	15	16	17	18	19	20	21	22	23	24	25	26	27	28	29	30	31	32	33	34	35
1. *M. borneensis*	**0.0**																																		
2. *Microhyla* sp. 1	3.0	–																																	
3. *M. malang*	2.6	3.6	**3.0**																																
4. *M. orientalis*	4.3	5.7	5.4	–																															
5*. M. mantheyi*	5.3	5.3	6.3	5.2	**1.0**																														
6. *M. fodiens * **sp. nov.**	11.4	11.6	11.3	11.2	10.8	**0.0**																													
7. *M. achatina*	6.4	7.7	7.4	5.9	6.1	10.1	**2.8**																												
8. *M. gadjahmadai*	6.2	6.9	6.9	6.4	5.5	11.3	5.3	**2.6**																											
9. *M. kodial*	6.9	7.7	8.0	7.1	6.6	11.4	7.4	7.5	**0.4**																										
10. *M. irrawaddy * **sp. nov.**	7.7	7.9	8.0	7.7	7.7	11.2	7.0	7.0	5.3	**0.0**																									
11. *M. heymonsi*	6.7	6.8	6.5	7.0	7.4	9.1	7.9	8.4	7.1	7.5	**2.3**																								
12. *M. mukhlesuri*	8.2	9.3	8.1	7.6	7.6	10.7	8.1	8.1	7.0	7.9	7.1	**1.3**																							
13. *M. fissipes*	7.5	8.1	7.7	6.7	6.9	10.3	7.7	7.5	6.5	7.1	6.7	2.4	–																						
14. *M. mymensinghensis*	7.9	8.7	8.0	8.1	7.7	10.7	7.5	8.3	6.9	7.4	6.8	4.2	3.1	**0.1**																					
15. *M. okinavensis*	8.1	8.7	8.2	6.9	6.9	9.7	6.9	6.7	7.7	8.1	7.1	6.1	4.9	6.4	–																				
16. *M. beilunensis*	7.0	8.2	7.2	6.6	6.5	9.3	7.0	6.7	6.6	6.4	6.4	4.7	3.6	4.7	3.2	**0.1**																			
17. *M. mixtura*	7.7	9.3	8.0	7.9	7.4	9.2	7.9	7.6	7.5	7.3	7.4	5.7	4.6	5.0	4.5	3.2	**0.8**																		
18. *M. berdmorei*	9.9	9.9	10.2	9.4	9.4	8.8	8.0	9.3	11.6	10.7	9.8	10.0	9.4	10.4	9.3	9.5	9.8	**1.6**																	
19. *M. pulchra*	11.6	11.0	10.7	11.0	10.6	10.3	8.7	9.9	11.4	11.0	9.6	9.7	9.5	9.9	8.9	8.5	9.9	7.9	**0.0**																
20. *M. nilphamariensis*	9.1	9.8	9.1	9.8	9.3	10.2	8.8	8.9	10.0	9.6	9.9	8.5	8.3	8.3	9.6	8.3	8.7	8.9	8.9	–															
21. *M. taraiensis*	10.5	10.8	10.9	11.8	10.1	11.4	11.5	10.8	10.8	11.4	11.3	10.0	9.1	9.7	11.4	10.2	10.3	10.4	10.5	5.1	–														
22. *M. ornata*	11.0	11.8	10.6	11.6	11.3	10.3	10.1	11.0	12.0	11.8	10.2	9.2	8.9	8.9	9.7	9.9	9.9	9.3	9.5	4.9	6.7	–													
23. *M. rubra*	9.9	11.2	10.6	10.5	10.2	11.4	9.7	10.0	11.2	11.2	9.9	9.3	9.2	10.2	10.1	9.9	10.1	9.5	9.7	6.1	7.1	7.8	**0.4**												
24. *M. mihintalei*	9.6	10.4	10.3	10.0	9.5	11.0	9.8	9.5	10.8	11.2	9.6	9.8	9.2	10.2	9.6	9.7	10.0	9.8	9.4	6.2	7.6	8.0	2.7	**0.1**											
25. *M. aurantiventris*	10.8	11.2	10.5	11.2	10.3	10.1	11.3	10.9	10.6	12.0	10.1	11.4	10.8	10.5	10.5	9.4	9.9	11.3	10.1	8.1	9.9	9.5	11.0	10.2	**0.0**										
26. *M. butleri*	10.9	11.4	10.5	11.2	11.0	12.2	10.9	11.3	11.1	11.5	10.6	10.8	10.3	11.2	11.3	9.6	10.8	10.9	10.8	9.6	11.0	11.3	9.5	10.1	6.6	**1.7**									
27. *M. superciliaris*	11.1	10.6	10.6	11.1	10.1	11.3	11.3	11.5	10.7	11.3	8.7	9.3	8.6	9.7	10.6	9.3	10.2	10.4	11.3	8.8	8.6	9.2	8.7	8.5	8.8	8.1	**0.2**								
28. *M. laterite*	11.8	11.8	11.9	11.6	11.1	10.8	11.6	11.8	12.7	13.1	11.1	11.2	10.8	11.6	12.2	11.3	11.2	11.3	12.4	10.6	12.2	11.0	11.4	11.5	8.4	11.1	9.7	**0.0**							
29. *M. sholigari*	11.6	11.6	11.8	11.6	11.0	10.8	11.6	12.0	12.3	12.9	10.8	10.6	10.8	11.4	12.5	10.9	11.4	10.2	11.6	9.4	10.2	10.0	10.0	10.5	9.4	10.5	9.5	4.5	**0.0**						
30. *M. palmipes*	11.5	12.3	11.4	12.3	11.8	11.1	12.0	11.1	11.4	10.4	9.7	9.4	8.7	10.2	10.5	9.3	11.2	12.0	11.0	9.1	10.2	9.1	10.1	10.3	10.0	10.5	9.3	12.0	11.5	**2.8**					
31. *M. petrigena*	11.3	12.3	11.6	11.5	11.0	11.8	11.1	11.2	10.5	11.1	10.2	10.9	10.5	10.9	11.4	9.9	10.9	10.2	11.3	10.2	11.0	10.8	10.5	10.1	10.8	11.0	9.7	12.6	11.6	10.0	**4.5**				
32. *M. perparva*	11.0	11.7	11.1	11.7	11.6	11.5	12.0	11.3	10.8	11.2	10.1	10.3	9.6	10.0	11.2	10.1	11.0	11.5	11.6	10.8	11.2	10.5	11.4	11.2	10.0	10.9	10.0	11.7	11.8	9.3	6.0	**4.3**			
33. *M. annectens*	10.8	11.2	11.4	11.6	11.3	11.5	11.6	12.1	11.9	11.4	10.7	11.7	10.7	11.6	12.2	11.1	11.9	9.5	11.8	10.9	12.1	11.1	11.0	11.6	10.9	10.7	10.7	12.8	11.7	11.1	7.2	7.4	**0.6**		
34. *M. marmorata*	8.9	10.1	9.2	10.3	10.6	11.0	10.3	10.2	11.1	10.8	9.7	10.3	9.3	9.7	10.5	9.5	10.2	10.3	10.8	9.3	10.5	9.3	9.7	10.6	10.3	9.7	10.0	10.8	11.0	9.9	6.8	6.3	5.2	–	
35. *Microhyla* sp. 2	7.5	8.1	8.4	7.5	7.5	11.2	6.6	7.4	5.5	2.0	8.4	8.2	7.7	7.7	8.7	7.4	8.3	10.9	11.4	9.8	12.0	12.0	11.2	10.6	11.8	12.0	12.3	12.2	12.3	11.3	11.5	11.6	11.8	11.0	**0.0**

### Taxonomy

Our field survey in Myanmar revealed two morphologically distinct species of microhylids, which belong to the genus *Microhyla* based on morphological and molecular evidence and could not be assigned to any currently recognized species (see below).

Both species were allocated to the genus *Microhyla* as they show the following diagnostic characters of the genus (Inger, 1989; Matsui et al., 2013; Parker, 1934): small to medium body size; narrow head; eyes small with circular pupil; lack of small spine-like projections of skin at heel and elbow; maxillary and vomerine teeth absent; snout less than twice diameter of eye; tongue obovate, entire and free at base; fingers without webbing; toes with basal webbing; palmar tubercles distinct; prominent inner and outer metatarsal tubercles on foot; supratympanic fold present; and, tympanum hidden under skin.

Our mtDNA genealogy analyses based on the 12S rRNA–16S rRNA 2 481 bp-long mtDNA fragment indicated that both species belong to the *M. achatina* species group (Figure 3). The stout-bodied *Microhyla* sp. from Kan Pauk represents a distinct lineage within the species group and is highly divergent in 16S rRNA gene sequences from any congener for which homologous sequences are available (*P*-distance≥8.8%). The slender-bodied *Microhyla* sp. from the Magway Division is a sister species of an undescribed *Microhyla* sp. 2 from Sagaing Division in northern Myanmar and closely related to *M. kodial*, inhabiting southern India (*P*-distance≥5.3%).

The phylogenetic position of *Microhyla* spp. from the Magway Division, together with the observed differences in mtDNA sequences, is congruent with evidence from diagnostic morphological characters (see “Comparisons” sections). These results strongly support our hypothesis that the newly discovered populations of *Microhyla* spp. from the Magway Division represent two previously unknown species, which we describe below.

*Microhyla fodiens* **sp. nov.**

**Figure 4:**
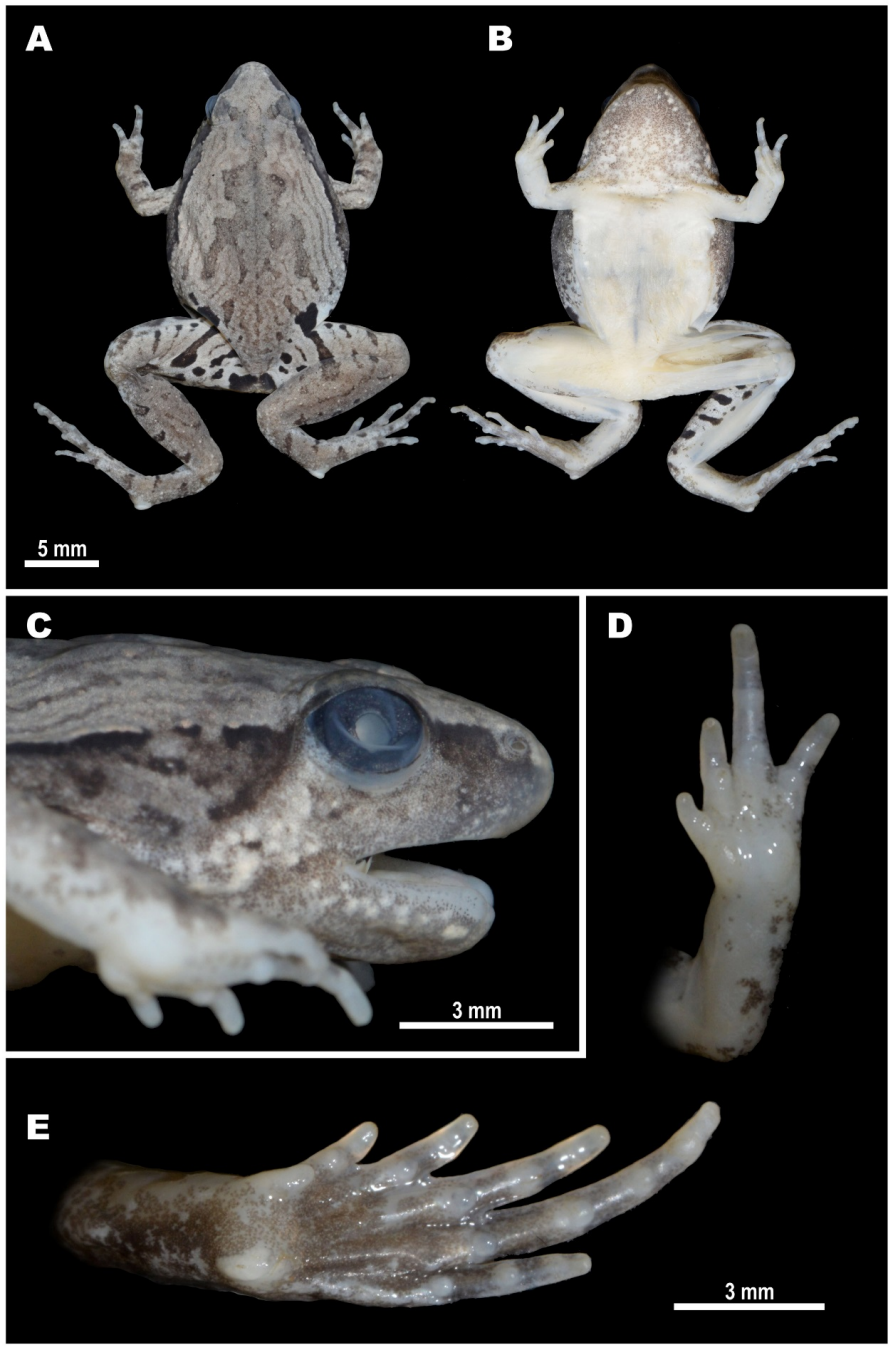
Holotype of *Microhyla fodiens* sp. nov. (ZMMU A5960), male, in preservative A: Dorsal view; B: Ventral view; C: Lateral view of head; D: Volar view of left hand; E: Plantar view of right foot. Photos by Nikolay A. Poyarkov.

**Figure 5:**
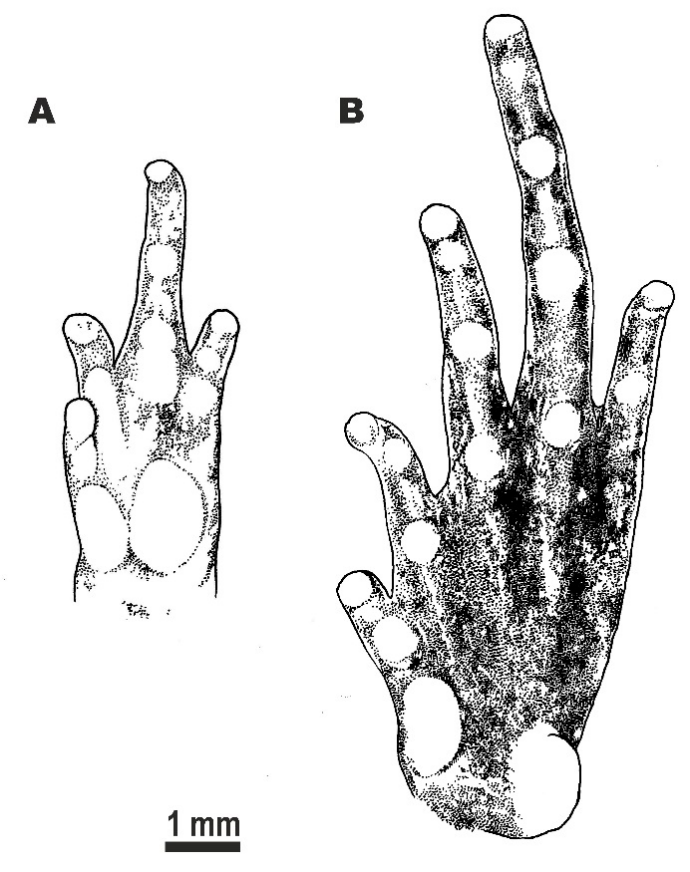
Morphological details of holotype of *Microhyla fodiens* sp. nov. (ZMMU A5960), male, in preservative A: Volar view of the left hand; B: Plantar view of left foot. Scale bar: 1 mm. Drawings by Valentina D. Kretova.

**Figure 6:**
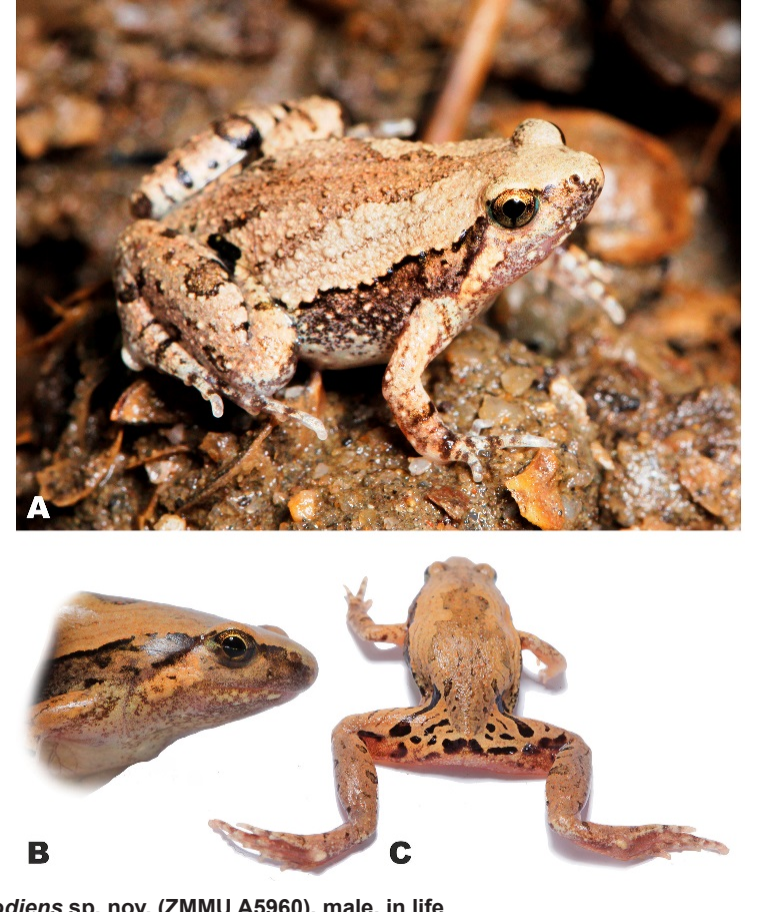
Holotype of *Microhyla fodiens* sp. nov. (ZMMU A5960), male, in life A: Dorsolateral view *in situ*; B: Lateral view of head; C: Posterior view of thighs and inguinal region showing regular black markings. Photos by Nikolay A. Poyarkov (A) and Parinya Pawangkhanant (B, C).

Table 3; Figures 2C, 4–6.

**Table 3:** Measurements of type series of *Microhyla fodiens *sp. nov. (in mm)

**No.**	**Specimen ID**	**Type status**	**SVL**	**HL**	**SL**	**EL**	**N-EL**	**HW**	**IND**	**IOD**	**UEW**	**FLL**	**LAL**	**HAL**	**1FL**	**IPTL**	**OPTL**	**3FDD**	**HLL**	**TL**	**FL**	**IMTL**	**1TOEL**	**4TDD**	**OMTL**
	**Male**																								
1	ZMMU A5960	Holotype	20.8	6.5	2.9	2.4	1.7	8.3	1.7	2.4	1.2	10.2	8.2	5.4	0.9	0.7	1.1	0.4	30.2	11.1	15.2	1.2	1.5	0.6	1.4
	**Sub adults**																								
2	ZMMU A5961	Paratype	17.9	5.4	2.6	2.2	1.4	7.5	1.4	2.0	1.3	9.4	7.2	4.8	0.8	0.8	1.0	0.4	25.5	9.2	13.0	1.0	1.5	0.5	1.1
3	ZMMU A5962	Paratype	15.9	5.6	2.3	2.3	1.4	6.7	1.4	1.9	1.1	7.4	6.6	4.2	0.8	0.6	0.9	0.3	22.4	8.5	11.3	0.8	1.5	0.4	0.8
4	ZMMU A5963	Paratype	15.1	5.0	2.4	2.2	1.3	6.3	1.4	1.8	1.2	8.9	6.7	4.3	0.8	0.7	0.9	0.4	24.1	8.0	11.0	0.9	1.4	0.6	0.8
5	ZMMU A5964	Paratype	14.5	4.7	2.3	1.9	1.4	5.5	1.5	2.0	1.1	8.0	6.3	4.0	0.9	0.7	0.8	0.4	22.5	8.1	11.0	0.8	1.5	0.4	1.0
6	ZISP 13729	Paratype	12.6	4.2	2.2	2.0	1.2	5.1	1.3	2.0	1.0	6.6	5.6	3.6	0.7	0.6	0.8	0.3	19.0	6.8	9.4	0.6	1.3	0.4	0.7
		**Mean**	16.1	5.2	2.4	2.2	1.4	6.2	1.5	2.0	1.2	8.4	6.8	4.4	0.8	0.7	0.9	0.4	23.9	8.6	11.8	0.9	1.5	0.5	0.9
		***SD***	*2.9*	*0.8*	*0.3*	*0.2*	*0.2*	*1.0*	*0.2*	*0.2*	*0.1*	*1.4*	*0.9*	*0.6*	*0.1*	*0.1*	*0.1*	*0.0*	*3.7*	*1.5*	*2.0*	*0.2*	*0.1*	*0.1*	*0.1*
		**Min**	12.6	4.2	2.2	1.9	1.2	5.1	1.3	1.8	1.0	6.6	5.6	3.6	0.7	0.6	0.8	0.3	19.0	6.8	9.4	0.6	1.3	0.4	0.7
		**Max**	20.8	6.5	2.9	2.4	1.7	7.5	1.7	2.4	1.3	10.2	8.2	5.4	1.1	0.8	1.1	0.4	30.2	11.1	15.2	1.2	1.7	0.6	1.1

Min: Minium; Max: Maximum. For other abbreviations see Materials and Methods.

### Chresonymy

*Microhyla rubra* – (?) Parker, 1934, p. 145 (B.M.87.2.26.24, coll. from “Moulmein, Burma” by W. Theobald).

*Microhyla rubra* – Wogan et al., 2008, p. 84–86; Peloso et al., 2016, p. 5, 23.

*Microhyla* sp. B – Mulcahy et al., 2018, p. 99, 116–117.

**Holotype:** ZMMU A5960 (field number NAP-08268), adult male collected on the bank of an artificial pond near a Buddhist pagoda in the small village of Kan Pauk in the vicinity of Shinma Taung Mt., Yesagyo Township, Magway Division, Myanmar (coordinates N21.595°, E95.074°; elevation 232 m a.s.l.), collected on 15 July 2018 at 1900 h by Nikolay A. Poyarkov, Vladislav A. Gorin, Parinya Pawangkhanant, and Than Zaw.

**Paratypes:** ZMMU A5961–A5964 (field numbers NAP-08269–08272) and ZISP 13729 (field number NAP-08273), five subadult specimens from the same locality and with the same collection information as the holotype.

**Referred specimens: **CAS 215851 (field number JBS-5249), collected from the same locality as the holotype on 16 August 2000 by H. Win, T. Thin, S.W. Kyi, and H. Tun.

**Diagnosis:** *Microhyla fodiens* **sp. nov.** is characterized by a combination of the following morphological attributes: (1) males with medium body size, SVL 20.8–29.12 mm in two adult individuals, body habitus stout; (2) head flattened, triangular, much wider than long, snout rounded in dorsal and bluntly rounded in lateral views, notably protruding above lower jaw in ventral aspect; *canthus rostralis* rounded, indistinct; (3) skin on dorsum and flanks feebly granular with numerous small round tubercles, ventral surfaces smooth; (4) dorsolateral skinfold presents as row of large tubercles ventrally underlined with black stripe; (5) mid-vertebral skin ridge and dorsomedial stripe absent; (6) supratympanic fold almost indistinct; (7) finger I well developed, notably less than one-half length of finger II; (8) finger and toe tips lacking disks and median longitudinal grooves; (9) two large palmar tubercles (inner palmar tubercle ovoid, slightly elongated; outer palmar tubercle almost rounded); (10) two very prominent metatarsal tubercles (inner metatarsal tubercle large, bean-shaped, outer metatarsal tubercle greatly enlarged, shovel-shaped); (11) limbs short, tibiotarsal articulation of adpressed limb not reaching eye level; (12) toe webbing basal, reaching proximal tubercles; webbing formula: I 1–2 II 1¾–3 III 2¾–3¾ IV 4–2¾ V; (13) superciliary tubercles absent; (14) dorsum beige-brown with “teddy-bear-shaped” dark-brown marking running from interorbital to sacral region; two large dark-black inguinal spots continuing on dorsal surfaces of thighs; posterior surfaces of thighs and cloacal region with regular black stripes; chin and throat marbled with gray, chest and belly whitish, limbs ventrally pink. Interspecific genetic *P*-distances in 16S rRNA gene fragment between new species and congeners vary from 9.1% to 12.4%.

**Description of holotype** (Figures 2C, 4–6): Medium-sized male specimen in good state of preservation, SVL 20.1 mm; habitus stout (Figure 4A), head small, much shorter than wide (HL/HW 78.6%); snout rounded in dorsal view (Figure 4A), bluntly rounded in lateral profile (Figure 4C), notably protruding above lower jaw in ventral view (Figure 4B), longer than eye diameter (EL/SL 83.8%); eye small, rounded, almost not protuberant in dorsal (Figure 4A) and lateral views (Figure 4C), pupil circular (Figure 4C); dorsal surface of head flattened, *canthus rostralis* indistinct, rounded; loreal region vertical, not concave; nostril rounded with lateral orientation, located much closer to tip of snout than to eye (Figure 4C); tympanum hidden under skin of temporal region, supratympanic fold smooth, weak, almost indistinct, running ventroposteriorly from posterior corner of eye to axilla; maxillary and vomerine teeth absent, tongue obovate, entire and free at base, lacking papillae; vocal sac single, subgular.

Forelimbs short, three times shorter than hindlimbs (FLL/HLL 33.9%); hand short, notably shorter than lower arm (HAL/LAL 65.5%) and two times shorter than forelimb length (HAL/FLL 52.8%); fingers short, thick, rounded in cross-section, first finger well developed, but two times shorter than second finger (1FL/2FL 46.9%); relative finger lengths: I<IV<II<III (see Figures 4D, 5A). Finger webbing and dermal fringes absent; tips of all fingers rounded, not enlarged, lacking terminal disks and median longitudinal furrows or grooves; subarticular tubercles on volar surface of fingers very large, distinct, rounded, prominent; finger subarticular tubercle formula: 1:1: 2:2 (hereafter, given for fingers I:II:III:IV, respectively); nuptial pad absent; two metacarpal (palmar) tubercles: inner palmar tubercle slightly elongated, ovoid-shaped, two times longer than wide; outer palmar tubercle flattened, large, rounded, notably longer than inner (IPTL/OPTL 81.8%); medial or supernumerary palmar tubercles absent; inner and outer palmar tubercles separated by deep groove.

Hindlimbs massive and comparatively short, tibia length slightly longer than half of snout-vent length (TL/SVL 53.4%), hindlimb length less than 1.5 times longer than snout-vent length (HLL/SVL 145.0%); tibiotarsal articulation of adpressed limb not reaching orbit level; foot slightly longer than tibia (FL/TL 137.2%); relative toe lengths: I<II<V<III<IV; tarsal fold on inner surface of tarsus absent; tips of all toes rounded, not enlarged, not forming terminal disks (Figures 4E, 5B); toes thick, short, slightly flattened in cross-section, with weak dermal fringes present on toes II–V reaching level of penultimate phalanges (Figures 4E, 5B); basal webbing developed between all toes, webbing formula: I 1–2 II 1¾–3 III 2¾–3¾ IV 4–2¾ V; subarticular tubercles on toes very distinct, protruding, rounded, toe subarticular tubercle formula: 1:2:3:3:2 (hereafter, given for toes I:II:III:IV:V, respectively); nuptial pad absent; two large metatarsal tubercles: inner metatarsal tubercle elongated, prominent, bean-shaped; outer metatarsal tubercle very large, shovel-shaped, with prominent outer edge (OMTL/IMTL 118.6%) (Figure 5B).

Dorsal skin feebly tubercular with numerous small granules and tubercles evenly scattered on dorsum; more distinct in life (Figure 6A) than in preservative (Figure 4A); upper eyelids almost smooth with few flat tubercles on medial edges of eyelids, superciliary tubercles or projections absent; mid-vertebral dermal ridge absent; indistinct dorsolateral skinfold running from posterior eye corner towards groin, consisting of row of larger glandular warts (Figure 6A); skin on dorsolateral surfaces smooth with rare small granules; dorsal surface of limbs smooth with few small tubercles, ventral sides of trunk, head, and limbs smooth.

**Coloration of holotype in life:** Dorsal surfaces of head and trunk with beige background (Figure 6); weak brown interorbital bar between eyelids posteriorly forming “teddy-bear”-like (see Rakotoarison et al., 2017) or hourglass-shaped dark-brown markings, running posteriorly to scapular region, widening at level of axilla, narrowing at mid-dorsum and widening again towards groin; thin brownish lines on lateral sides of dorsum forming three nested reverse V-shaped figures, parallel to edges of “teddy-bear”-like dark marking; sacral region with irregular brownish vermiculated pattern; two large dark-black inguinal spots in groin area continuing posteriorly on dorsal surfaces of thighs, forming thick dark-black cross-bands (Figure 6A). Dorsolateral lines ventrally edged with black; body flanks with numerous small blackish and dark-brown spots and mottling; ventral edge of eyelids dark-brown; lateral sides of head with weak brownish mottling; supratympanic dark-brown with light yellowish-beige stripe ventrally, running from posterior edge of eye towards axilla; dorsal surfaces of limbs beige-brown with few brownish blotches; two narrow dark-brown bars on dorsal surfaces of forearm on each forelimb; three wide black transverse interrupted cross-bands on dorsal and posterior surfaces of proximal part of thighs forming tiger-like pattern, cloacal region with large dark-black blotch (Figure 6C); dorsal surfaces of tibia and tarsus brownish with rare dark transverse blotches alternating on each hindlimb: four large spots on right shank, three large dark spots on left shank; two dark short stripes on each tarsus (Figure 6A, C); fingers and toes dorsally gray with brown cross-bars; throat and chest with gray mottling, chest and venter whitish; ventral surfaces of limbs pinkish to gray-violet; hand and foot ventrally pinkish-gray; pupil black, circular, edged with narrow golden line, dense golden reticulations throughout iris except for dark vertical bar at ventral part of iris; sclera bluish (Figure 6A, B).

**Coloration of holotype in preservative:** After initial fixation in formalin and preservation in ethanol for six months, dorsal coloration significantly faded and turned light grayish-brown (Figure 4A), ventral surface of chest, belly, and limbs changed to whitish (Figure 4B); dorsal pattern and dark stripes on dorsal surfaces of limbs and body unchanged; iris coloration faded and turned dark-gray (Figure 4C).

**Measurements of holotype (in mm):** SVL 20.8; HL 6.5; SL 2.9; EL 2.4; N-EL 1.7; HW 8.3; IND 1.7; IOD 2.4; UEW 1.2; FLL 10.2; LAL 8.2; HAL 5.4; IPTL 0.7; OPTL 0.9; 3FDD 0.4; HLL 30.2; TL 11.1; FL 15.2; IMTL 1.2; 4TDD 0.6; OMTL 1.4; 1FL 0.9; 2FL 1.9; 3FL 2.9; 4FL 1.6; 1TOEL 1.5; 2TOEL 2.6; 3TOEL 3.8; 4TOEL 5.5; 5TOEL 2.8.

**Variation**: Morphometric variation of the type series is presented in Table 3. The paratypes are subadult specimens and are notably smaller in body size than the holotype (SVL 12.6–17.9 mm; mean 15.2±1.38 mm; *n*=5). Paratype coloration does not significantly differ from that described for the holotype, with the exception of the throat, which is off-white and lacks blackish mottling. All type specimens have large black inguinal spots; location and shape of black markings on posterior surfaces of thighs and cloacal area, as well as shape of “teddy-bear”-shaped brown dorsal marking, may vary insignificantly. Adult male CAS 215851 (see Referred materials) from the type locality is larger than the holotype (SVL 29.1 mm) but agrees well with the holotype description in general morphology and coloration, although it has more dark spots in the axilla area compared to the holotype. CAS 215851 has a very large and shovel-shaped outer metatarsal tubercle, notably protruding in dorsal view. Chromatic differences include coloration of the throat, which is uniform black-gray in male CAS 215851; coloration gets darker towards lower jaw edges.

**Natural history:** Pakokku District is located in the heart of the dry zone of central Myanmar, which is characterized by low precipitation and high temperatures, with a hot semi-arid tropical savanna-like climate (Peel et al., 2007). On average, Pakokku receives around 560 mm of precipitation annually. April is the warmest month, with an average temperature of 31.5 °C, whereas January is the coldest month, with an average temperature of 21.5 °C. The highest rainfall is observed in August and September, with 113 and 130 mm of precipitation, respectively (data fromhttps: //en.climate-data.org).

**Figure 7:**
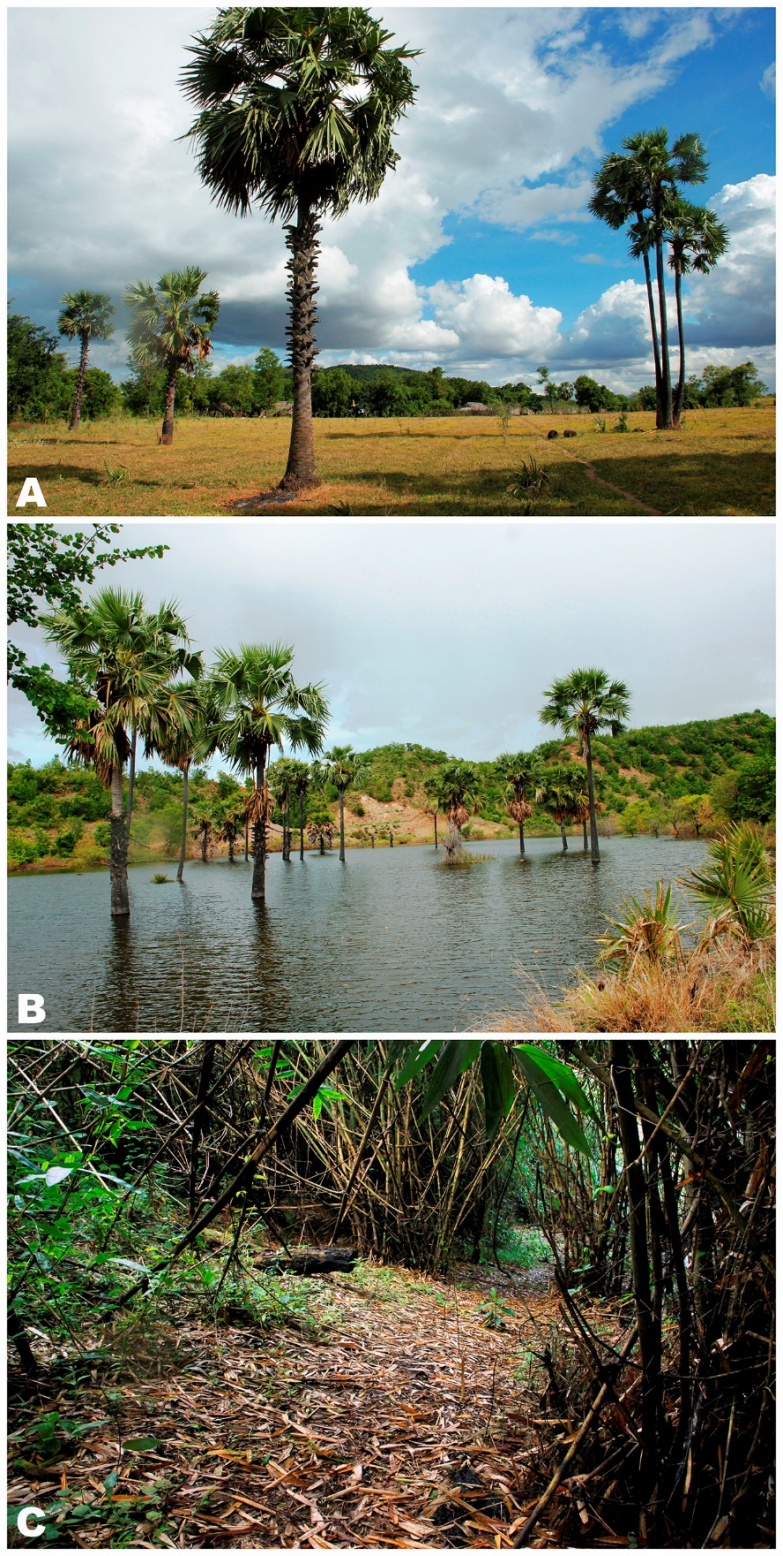
Natural habitats of *Microhyla* in Myanmar A: Natural habitat of *Microhyla fodiens* **sp. nov.** at Kan Pauk village, Magway (type locality), green hill in background is Shinma Taung Mountain; B: Breeding habitat of *Microhyla irrawaddy* **sp. nov.** from Pakokku, Magway (type locality); C: Natural habitat of *M. heymonsi*, *M. butleri*, and *M. mukhlesuri* in bamboo forest on slopes of Ingyin Taung Mt., Indawgyi Lake region, Kachin. Photos by Parinya Pawangkhanant.

All specimens of *Microhyla fodiens* **sp. nov.** were collected at night from 1900 to 2100 h on the banks of a large, permanent, likely artificial pond near a small Buddhist pagoda in the center of Kan Pauk village, located in a dry and open habitat with rare vegetation in the vicinity of Shinma Taung Mountain – the only hill in the area with an elevation of 514 m a.s.l. (Figure 7A). The pond is used by local people as a watering area for livestock. Subadult specimens were recorded on the banks of the pond hiding in cracks, whereas the adult male holotype was collected from the stone fence of the pagoda, hiding in a crevice. Thus, the new species appears to be a good burrower. During the survey, the weather remained hot and dry and the *Microhyla fodiens* **sp. nov.** specimens were inactive and hid in shelters; no calling was recorded. We also examined several paddy-fields and other large waterbodies within a 2 km radius around Kan Pauk village; however, *Microhyla fodiens* **sp. nov.** was not recorded in any other locality. Diet, larval stages, and eggs of the new species are unknown.

Other microhylids recorded in sympatry with *Microhyla fodiens* **sp. nov.** included new congeneric species *Microhyla irrawaddy* **sp. nov.** (see below) and *Kaloula pulchra* Gray, 1831, which appear to share the same breeding site during the reproductive season. Other anurans such as *Fejervarya* sp., *Hoplobatrachus* cf. *tigerinus* (Daudin, 1802), *Sphaerotheca* sp., and *Duttaphrynus melanostictus* (Schneider, 1799) also occurred in sympatry*.*

**Distribution:** *Microhyla fodiens* **sp. nov.** is currently known only from the type locality in Kan Pauk, Yesagyo Township, Magway Division, Myanmar (Figure 1). The species was recorded at an elevation of 230 m a.s.l. The actual distribution of the new species is unknown, but it is likely to be found in other arid areas of the Irrawaddy River Valley in the region of the Irrawaddy and Chindwin interfluve; discovery of new localities in Magway, Sagaing, and Mandalay divisions is anticipated. The record of “*Microhyla rubra*” from “Moulmein, Burma” (now Mawlamyine) by Parker (1934) based on W. Theobald’s collection comes from Mon State in southern Myanmar—a region with a much milder tropical monsoon climate—might refer to a different species. The taxonomic status of this record requires clarification by further studies.

**Conservation status:** At present, the new species is known from several specimens from a single locality in Yesagyo Township, Magway Division; however, a wider distribution in other arid areas of central Myanmar is anticipated. As the actual range and population trend of the new species are currently unknown, we suggest *Microhyla fodiens* **sp. nov.** be considered as a Data Deficient species following IUCN’s Red List categories (IUCN Standards and Petitions Subcommittee, 2017).

**Etymology:** The specific name “*fodiens*” is a Latin adjective in the nominative singular derived from “*fodio*” — Latin verb meaning “to dig” or “to burrow” referring to the distinctive enlarged shovel-shaped outer metatarsal tubercle of the new species, suggesting that it is a good burrower, which may serve as an adaptation to the dry climate of the Irrawaddy River Valley in central Myanmar. The recommended common name in English is “*Burrowing narrow-mouth frog*”. The recommended common name in Burmese is “*Twin Aoung Thaephar*”.

**Comparisons:** Only a few species of *Microhyla* have a stout body habitus with an enlarged spade- or shovel-shaped outer metatarsal tubercle as an adaptation for digging, including, *M. rubra* from southern India, *M. mihintalei* from Sri Lanka, *M. taraiensis* from eastern Nepal, and *M. picta* from southern Vietnam. Comparisons of *Microhyla fodiens* **sp. nov.** with the abovementioned species appear to be the most pertinent; from all remaining species of the genus, the new species can be easily distinguished by its stout body habitus and enlarged shovel-shaped outer metatarsal tubercle (vs. slender to stout body habitus and small or no outer metatarsal tubercle in other species of *Microhyla*).

*Microhyla rubra* was originally described by Jerdon (1854) from “Carnatic near rivers” and “also Ceylon”; the holotype is considered to be lost. Recently, Wijayathilaka et al. (2016) restricted the distribution of *M. rubra* to southern India and Garg et al. (2018b) designated a neotype for this species. *Microhyla fodiens* **sp. nov.** can be distinguished from *M. rubra* from southern and eastern India by the following characteristics: first finger notably shorter than half of second finger (vs. equal), thigh shorter than foot length, TL=8.6±1.5 mm, FOL=11.8±2.0 mm, *n*=6 (vs. thigh longer than foot length, male, TL 13.8±0.5 mm, FOL 12.4±0.4 mm, *n*=8; data from Wijayathilaka et al., 2016), comparatively shorter hindlimbs with tibiotarsal articulation not reaching eye level (vs. reaching over eye level but shorter than snout tip), comparatively better developed webbing between toes, toe webbing formula: I 1–2 II 1¾–3 III 2¾–3¾ IV 4–2¾ V (vs. I 1½–2 II 1½–3 III 2½–3 IV 4–2½ V), and dorsal pattern with brown “teddy-bear”-shaped marking, thin brownish lines on lateral sides of dorsum forming three nested reverse V-shaped figures (vs. almost uniform reddish-brown dorsum).

*Microhyla fodiens* **sp. nov.** can be distinguished from *M. mihintalei* from Sri Lanka by the following characteristics: granular skin on dorsum (vs. shagreened or sparsely granular skin on dorsum), thigh shorter than foot length, TL=8.6±1.5 mm, FOL=11.8±2.0 mm, *n*=6 (vs. thigh equal to foot length, male, TL 11.6±0.6 mm, FOL 11.6±0.6 mm, *n*=14; data from Wijayathilaka et al., 2016), comparatively better developed foot webbing (vs. toe webbing reduced), dorsal pattern with brown “teddy-bear”-shaped marking, thin brownish lines on lateral sides of dorsum forming three nested reverse V-shaped figures (vs. almost uniform orange-brown or reddish-brown dorsum), and three wide black transverse cross-bands on dorsal and posterior surfaces of thighs forming tiger-like pattern, cloacal region with large black blotch (vs. tiger-like pattern and dark cloacal blotch absent).

The new species can be readily diagnosed from *M. taraiensis* from eastern Nepal by the following characteristics: red spots and tubercles on dorsum absent (vs. light red dots dispersed over dorsal surfaces), large shovel-shaped outer metatarsal tubercle (vs. rounded outer metatarsal tubercle), second finger longer than fourth finger (vs. shorter), comparatively shorter hindlimbs with tibiotarsal articulation not reaching eye level (vs. reaching nostril level), inner palmar tubercle ca. 1.5 times smaller than outer palmar tubercle (vs. inner palmar tubercle two times greater than outer palmar tubercle), single subarticular tubercle on second finger and two subarticular tubercles on third finger (vs. two tubercles on second finger and three tubercles on third finger), and single subarticular tubercle on second toe and two subarticular tubercles on third toe (vs. two tubercles on second toe and three tubercles on third toe).

Finally, *Microhyla fodiens* **sp. nov.** can be distinguished from *M. picta* from southern Vietnam by the following characteristics: generally smaller body size, adult SVL 20.8–29.1 mm (vs. adult SVL 25.2–33.4 mm), better developed webbing on feet, toe webbing formula: I 1–2 II 1¾–3 III 2¾–3¾ IV 4–2¾ V (vs. I 2–2¾ II 1¾–2¾ III 2¾–3¾ IV 4–2½ V), dorsal pattern with brown “teddy-bear”-shaped marking, thin brownish lines on lateral sides of dorsum forming three nested reverse V-shaped figures (vs. brown dorsal markings in shape of irregular blotches or reverse V-shaped figures but always edged with white or light beige), pronounced dorsolateral fold as row of enlarged tubercles (vs. no dorsolateral fold), bright-yellow coloration in groin area absent (vs. present), and black iris with dense golden reticulations (vs. dark iris with bronze to reddish-bronze color of reticulations).

*Microhyla irrawaddy* **sp. nov.**

**Figure 8:**
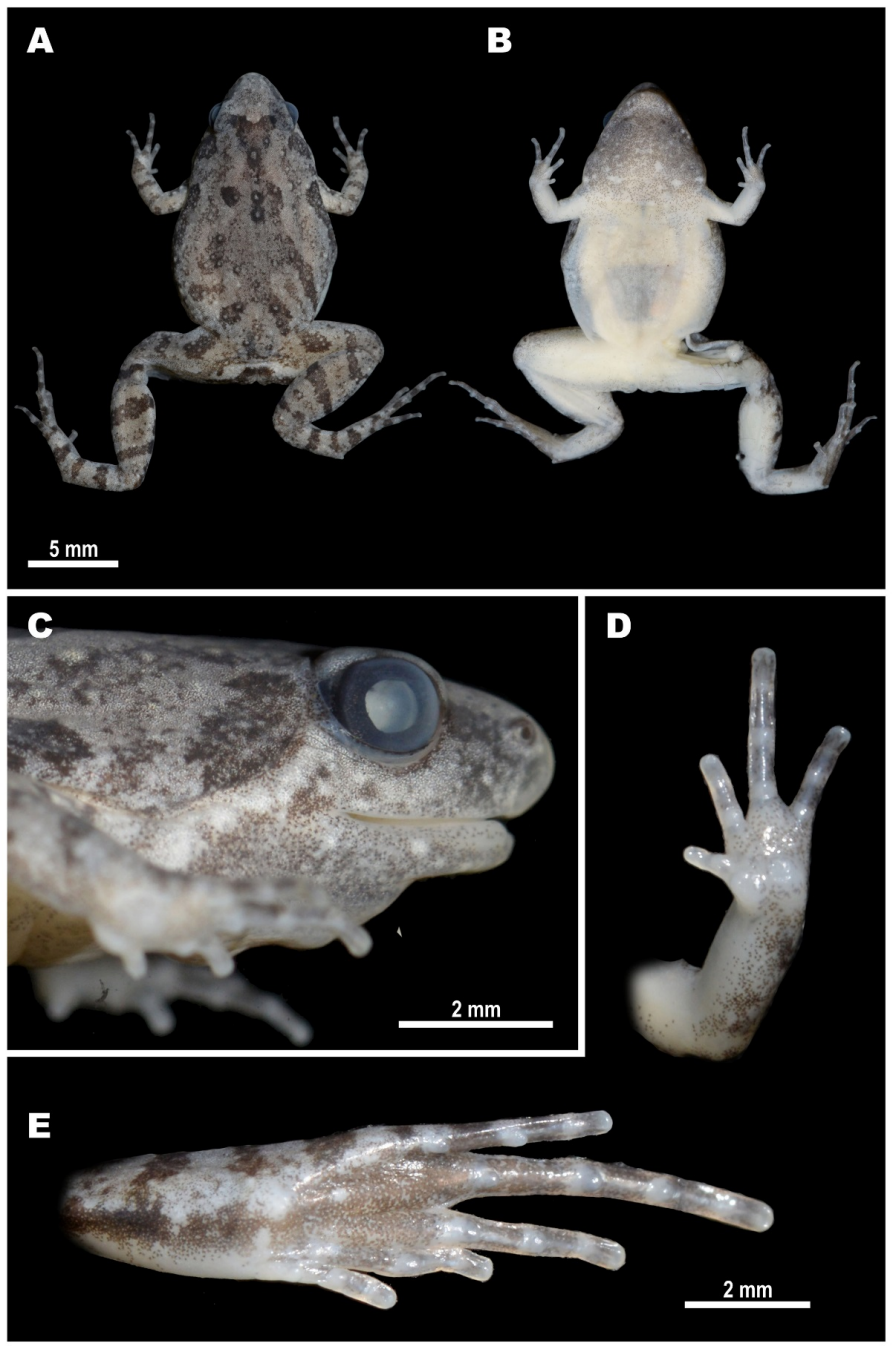
Holotype of *Microhyla irrawaddy *sp. nov. (ZMMU A5965), male, in preservative A: Dorsal view; B: Ventral view; C: Lateral view of head; D: Volar view of left hand; E: Plantar view of right foot. Photos by Nikolay A. Poyarkov.

**Figure 9:**
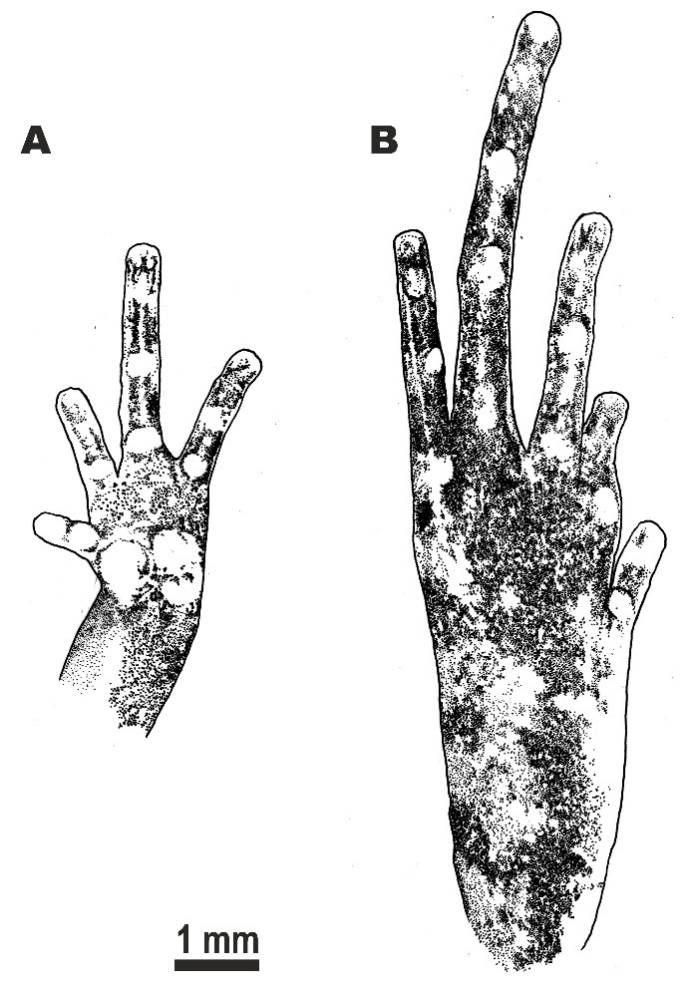
Morphological details of holotype of *Microhyla irrawaddy *sp. nov. (ZMMU A5965), male, in preservative A: Volar view of left hand; B: Plantar view of right foot. Scale bar: 1 mm. Drawings by Valentina D. Kretova.

**Figure 10:**
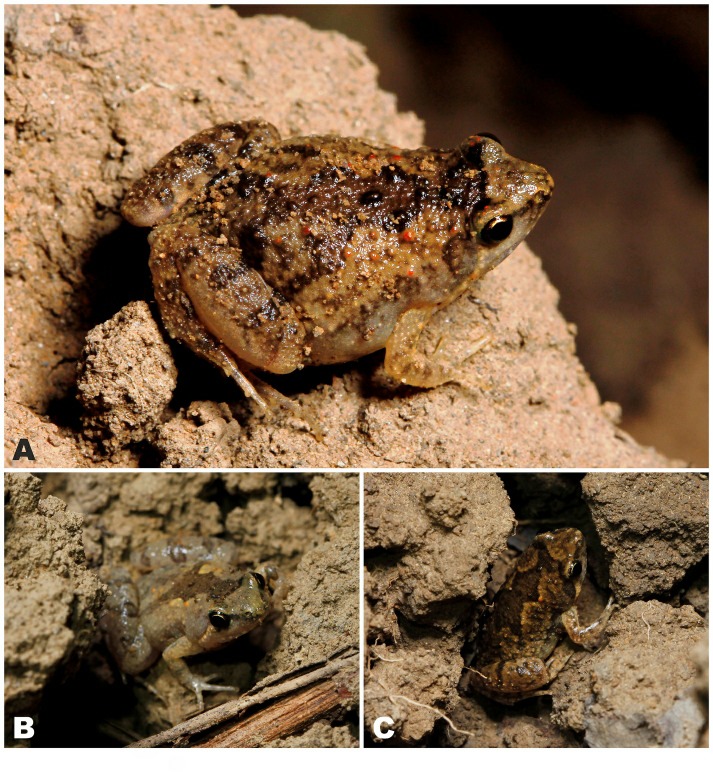
*Microhyla irrawaddy *sp. nov. type series *in situ* A: Dorsolateral view of holotype (ZMMU A5965); B, C: Paratype males (ZMMU A5966 and A5967) in calling position in hollows and buffalo footprints in dirt at type locality. Photos by Parinya Pawangkhanant.

Table 4; Figures 2A–B, 8–10.

**Table 4:** Measurements of type series of *Microhyla irrawaddy *sp. nov. (in mm)

**No.**	**SpecimenID**	**Typestatus**	**SVL**	**HL**	**SL**	**EL**	**N-EL**	**HW**	**IND**	**IOD**	**UEW**	**FLL**	**LAL**	**HAL**	**1FL**	**IPTL**	**OPTL**	**3FDD**	**HLL**	**TL**	**FL**	**IMTL**	**1TOEL**	**4TDD**	**OMTL**
	**Males**																								
1	ZMMU A5965	Holotype	15.6	5.1	2.4	2.1	1.4	6.9	1.3	1.8	1.1	7.7	6.3	3.9	0.9	0.6	0.7	0.4	22.6	7.9	11.5	0.7	1.5	0.4	0.3
2	ZMMU A5966	Paratype	17.1	5.3	2.4	2.3	1.3	6.9	1.3	1.9	1.2	7.8	6.6	3.9	0.9	0.6	0.6	0.3	23.6	8.0	11.2	0.6	1.4	0.4	0.3
3	ZMMU A5967	Paratype	15.4	4.9	2.5	2.2	1.2	7.0	1.3	1.9	1.2	7.6	6.0	3.7	0.7	0.5	0.6	0.3	22.9	7.7	10.9	0.7	1.2	0.3	0.4
4	ZMMU A5968	Paratype	12.4	4.1	1.7	1.7	1.2	5.1	1.3	1.5	1.1	6.8	5.5	3.4	0.8	0.3	0.6	0.3	18.9	6.7	10.1	0.6	1.3	0.3	0.2
5	ZMMU A5969	Paratype	16.0	5.1	2.3	2.2	1.3	6.2	1.3	1.8	1.1	8.2	7.1	4.5	0.9	0.6	0.7	0.4	24.3	8.3	12.2	0.6	1.6	0.4	0.4
6	ZMMU A5970	Paratype	15.3	5.2	2.3	1.8	1.1	6.7	1.3	1.7	1.3	8.5	6.9	4.2	0.8	0.5	0.7	0.3	22.6	7.9	11.3	0.5	1.4	0.3	0.4
7	ZISP 13730	Paratype	12.3	4.1	1.8	1.7	1.1	4.7	1.3	1.4	0.9	6.6	5.2	3.5	0.8	0.4	0.6	0.3	18.6	6.5	9.7	0.5	1.3	0.4	0.2
		**Mean**	14.9	4.8	2.2	2.0	1.2	6.2	1.3	1.7	1.1	7.6	6.2	3.9	0.8	0.5	0.7	0.3	21.9	7.6	11.0	0.6	1.4	0.4	0.3
		***SD***	*1.8*	*0.5*	*0.3*	*0.3*	*0.1*	*1.0*	*0.0*	*0.2*	*0.1*	*0.7*	*0.7*	*0.4*	*0.1*	*0.1*	*0.1*	*0.0*	*2.2*	*0.7*	*0.8*	*0.1*	*0.1*	*0.1*	*0.1*
		**Min**	12.3	4.1	1.7	1.7	1.1	4.7	1.3	1.4	0.9	6.6	5.2	3.4	0.7	0.3	0.6	0.3	18.6	6.5	9.7	0.5	1.2	0.3	0.2
		**Max**	17.1	5.3	2.5	2.3	1.4	7.0	1.3	1.9	1.3	8.5	7.1	4.5	0.9	0.6	0.7	0.4	24.3	8.3	12.2	0.7	1.6	0.4	0.4
	**Females**																								
8	ZMMU A5971	Paratype	16.7	5.1	2.0	2.2	1.2	7.2	1.4	1.9	1.0	7.5	6.2	3.6	1.0	0.6	0.9	0.3	23.3	8.0	11.9	0.6	1.6	0.4	0.4
9	ZMMU A5972	Paratype	17.6	5.3	2.5	2.2	1.4	7.7	1.6	2.1	1.2	9.5	7.4	4.7	1.1	0.7	0.7	0.4	26.7	9.4	13.3	0.6	1.8	0.4	0.4
10	ZMMU A5973	Paratype	17.7	5.4	2.2	2.2	1.3	6.7	1.5	2.0	1.1	9.2	6.6	4.8	1.0	0.6	0.7	0.4	26.5	9.2	12.6	0.4	1.7	0.4	0.4
11	ZMMU A5974	Paratype	17.5	5.3	2.2	2.2	1.4	7.7	1.6	2.1	1.4	9.2	7.4	4.8	0.9	0.6	0.8	0.4	25.4	8.9	13.0	0.6	1.6	0.5	0.5
12	ZMMU A5975	Paratype	20.9	6.3	2.5	2.4	1.5	7.8	1.5	2.1	1.3	9.5	7.4	4.8	1.0	0.7	1.0	0.4	28.1	10.0	14.1	0.7	1.6	0.5	0.5
13	ZMMU A5976	Paratype	18.2	5.4	2.3	2.2	1.3	7.4	1.5	2.0	1.3	9.2	7.6	4.5	0.9	0.7	0.8	0.5	24.7	8.8	12.2	0.6	1.6	0.5	0.4
		**Mean**	18.1	5.5	2.3	2.2	1.3	7.4	1.5	2.0	1.2	9.0	7.1	4.5	1.0	0.6	0.8	0.4	25.8	9.0	12.8	0.6	1.7	0.4	0.4
		***SD***	*1.5*	*0.4*	*0.2*	*0.1*	*0.1*	*0.4*	*0.1*	*0.1*	*0.1*	*0.8*	*0.6*	*0.4*	*0.1*	*0.1*	*0.1*	*0.0*	*1.7*	*0.7*	*0.8*	*0.1*	*0.1*	*0.1*	*0.1*
		**Min**	16.7	5.1	2.0	2.2	1.2	6.7	1.4	1.9	1.0	7.5	6.2	3.6	0.9	0.6	0.7	0.3	23.3	8.0	11.9	0.4	1.6	0.4	0.4
		**Max**	20.9	6.3	2.5	2.4	1.5	7.8	1.6	2.1	1.4	9.5	7.6	4.8	1.1	0.7	1.0	0.5	28.1	10.0	14.1	0.7	1.8	0.5	0.5

Min: Minium; Max: Maximum. For other abbreviations see Materials and Methods.

### Chresonymy

*Microhyla* sp. A – (?) Mulcahy et al., 2018, p. 99, 116–117.

**Holotype:** ZMMU A5965 (field number NAP-08241), adult male collected while calling from holes/hollows in the bank of a temporary pond in the Irrawaddy (Ayeyarwady) River Valley, in the suburbs of Pakokku, Pakoku District, Magway Division, Myanmar (coordinates N21.316°, E95.053°; elevation 59 m a.s.l.), collected on 14 July 2018 at 1900 h by Nikolay A. Poyarkov, Vladislav A. Gorin, Parinya Pawangkhanant, and Than Zaw.

**Paratypes:** ZMMU A5966–A5970 (field numbers NAP-08238–08240; NAP-08242–08243), and ZISP 13730 (field number NAP-08244), six adult males from the same locality and with the same collection information as the holotype; ZMMU A5971–A5974 (field numbers NAP-08245–08248), four adult females from the same locality and with the same collection information as the holotype; ZMMU A5975–A5976 (field numbers NAP-08274–08275), two adult females collected on the bank of a paddy field in the vicinity of Kan Pauk village, near Shinma Taung Mt., Yesagyo Township, Magway Division, Myanmar (coordinates N21.594°, E95.082°; elevation 217 m a.s.l.), collected on 15 July 2018 at 1900 h by Nikolay A. Poyarkov, Vladislav A. Gorin, Parinya Pawangkhanant, and Than Zaw.

**Diagnosis:** *Microhyla irrawaddy* **sp. nov.** is distinguished by the following combination of morphological characters: (1) small adult body size: males SVL 12.3–17.1 mm, females SVL 16.7–20.9 mm, body habitus very slender; (2) head small, triangular, wider than long, snout acuminate with rounded tip in dorsal view and rounded in lateral view, slightly protruding above lower jaw in ventral aspect; *canthus rostralis* indistinct; (3) skin on dorsum and flanks granular with irregularly scattered numerous large and small round tubercles, ventral surfaces completely smooth; (4) dorsolateral skinfold and dark lateral band absent; (5) mid-vertebral skin ridge and dorsomedial stripe absent; (6) supratympanic fold distinct; (7) finger I well developed, slightly longer than one-half length of finger II; (8) tips of fingers II–IV and toes II–V weakly dilated, not forming conspicuous disks; peripheral grooves ventrally present on tips of fingers II–IV and toes II–IV; fingers and toes lacking dorsal median grooves or distal notches; (9) two small palmar tubercles (inner palmar tubercle rounded, prominent; outer palmar tubercle smaller and less distinct than inner, rounded, flattened); (10) two small metatarsal tubercles (inner metatarsal tubercle elongated, ovoid, flattened; outer metatarsal tubercle small, rounded, prominent); (11) limbs comparatively short, tibiotarsal articulation of adpressed limb reaching eye level; (12) toe webbing completely reduced; webbing formula: I 2–3 II 2–3 III 3–4½ IV 4½–2¾ V; (13) superciliary tubercles absent; (14) dorsum yellowish-brown with dark-brown contrasting “teddy-bear”-shaped marking running from interorbital to sacral region; larger tubercles on dorsum orange to red; body flanks grayish with darker mottling not clearly separated from dorsum coloration; dorsal surfaces of thighs and shanks with two to three dark crossbars; chin and throat with grayish mottling (blackish in males), body and limbs ventrally cream to whitish at belly. Interspecific genetic *P*-distances in the 16S rRNA gene fragment between the new species and other currently recognized species of *Microhyla* vary from 5.7% to 12.9%.

**Description of holotype** (Figures 8–9, 10A): Small-sized male specimen in good state of preservation, SVL 15.6 mm; habitus very slender (Figure 8A), head small, notably shorter than wide (HL/HW 73.3%); snout acuminate with rounded tip in dorsal view (Figure 8A), gently rounded in lateral profile (Figure 8C), slightly projecting above lower jaw in ventral aspect (Figure 8B); snout longer than eye diameter (EL/SL 87.9%); eyes rounded, notably protuberant in dorsal (Figure 8A) and lateral views (Figures 8C, 10A), pupil circular (Figure 8C); head dorsally flattened, *canthus rostralis* rounded, indistinct; loreal region slightly concave; nostril lateral, rounded, located much closer to tip of snout than to eye (Figure 8C); tympanum hidden, supratympanic fold distinct, prominent, gently curving ventroposteriorly from posterior corner of eye towards forelimb insertion; maxillary and vomerine teeth absent, tongue obovate with pointed tip, smooth margins, lingual papillae absent; vocal sac single, subgular.

Forelimbs comparatively short, three times length of hindlimbs (FLL/HLL 33.9%); hand short, shorter than lower arm (HAL/LAL 62.0%), two times forelimb length (HAL/FLL 51.2%); fingers comparatively long and thin, rounded in cross-section, first finger well developed, notably longer than half of second finger length (1FL/2FL 56.0%); relative finger lengths: I<II=IV<III (see Figures 8D, 9A). Finger webbing and dermal fringes absent; tip of finger I rounded, not enlarged, lacking terminal disk and median longitudinal furrow; tips of fingers II–IV slightly dilated, not forming conspicuous disks and lacking dorsal median grooves; peripheral grooves ventrally present on tips of fingers II–IV (Figures 8D, 9A). Subarticular tubercles on volar surface of fingers rounded, with indistinct borders, rather flattened, finger subarticular tubercle formula: 1:1:2:2; nuptial pad absent; two palmar tubercles: inner palmar tubercle rounded, slightly prominent, with distinct borders; outer palmar tubercle flattened, large, rounded, with indistinct borders, larger than inner (IPTL/OPTL 83.8%); supernumerary palmar tubercles absent.

Hindlimbs comparatively short and thin, tibia length equal to half of snout-vent length (TL/SVL 50.7%), hindlimb length around 1.5 times longer than snout-vent length (HLL/SVL 145.0%); tibiotarsal articulation of adpressed limb reaching eye level; foot notably longer than tibia (FL/TL 144.9%); relative toe lengths: I<V<II<III<IV; tarsal fold absent; tip of toe I rounded, not forming terminal disk; tips of toes II–V weakly dilated, not forming conspicuous disks; peripheral grooves ventrally present on toe tips II–IV (Figures 8E, 9B); toes thin, long, rounded in cross-section, lacking dermal fringes (Figures 8E, 9B); webbing completely reduced between all toes, webbing formula: I 2–3 II 2–3 III 3–4½ IV 4½–2¾ V; subarticular tubercles on toes distinct, rounded, protruding, toe subarticular tubercle formula: 1:1:2:3:2; nuptial pad absent; two small metatarsal tubercles, inner metatarsal tubercle elongated, ovoid, flattened; outer metatarsal tubercle small, rounded, prominent, around one third length of inner metatarsal tubercle (OMTL/IMTL 35.6%).

Dorsal skin granular with numerous small granules and larger tubercles irregularly scattered on dorsum; distinct in life (Figure 10A) as in preservative (Figure 8A); upper eyelids with numerous small tubercles scattered medially; superciliary tubercles or projections absent; mid-vertebral dermal ridge and dorsolateral folds absent; skin on dorsolateral surfaces with smaller flattened granules and pustules; dorsal surface of forearms, thighs, shanks, and tarsus with evenly scattered small tubercles; skin on ventral sides of trunk, head, and limbs smooth.

**Coloration of holotype in life:** Head and trunk dorsally with yellowish-brown background coloration (Figure 10A); dark-brown interorbital bar between eyelids forming V-shaped marking oriented posteriorly towards scapular region; dark-brown markings on dorsum “teddy-bear”-like shape: narrowing at head basis, widening to diamond-shape on scapular region (four blackish round spots forming cross), narrowing posteriorly, forming two pairs of dark stripes towards groin and sacral area. Indistinct brownish lines and blotches on sacral area and axillary region. Gray-brownish line with unclear edges running from posterior corner of eye along upper flanks toward groin. Thin dark-brown stripe from anterior corner of eye along *canthus rostralis* toward nostril. Lateral sides of head and trunk grayish to yellowish-gray (Figure 10A). Limbs dorsally yellowish-brown with darker brownish markings alternating on each limb: two brown cross-bars on forearms, brownish spots on dorsal surfaces of hands and fingers; two dark-brown blotches on dorsal surfaces of thighs, distal one continuing to shanks; shanks dorsally with three dark-brown cross-bars; dorsal surface of tarsus with two brown cross-bars; feet and toes with brownish spots. Ventral surfaces of chest and belly pale cream; throat with dense dark-gray mottling, getting darker toward margins of lower jaw and lower margin of upper jaw; light yellow pigmentation at junction of forelimbs; limbs ventrally pinkish. Tubercles and granules on dorsal surfaces of body, head, and thighs orange to bright-red (Figure 10A); superciliary area of upper eyelids lighter than medial area; supratympanic fold brown; light yellowish-cream stripe from posterior corner of eye toward forelimb insertion; cloacal region brownish. Pupil black, circular, edged with golden line, dense bronze-green to golden reticulations throughout iris; sclera greenish-yellow (Figure 10A).

**Coloration of holotype in preservative:** After preservation in formalin and storage in ethanol for six months, coloration of dorsal surfaces faded to grayish-brown (Figure 8A), ventral surface of chest, belly, and limbs changed to whitish-gray (Figure 8B). Dorsal pattern, brownish markings on dorsal surfaces of limbs and body unchanged; iris coloration completely faded to black (Figure 8C).

**Measurements of holotype (in mm):** SVL 15.6; HL 5.1; SL 2.4; EL 2.1; N-EL 1.4; HW 6.9; IND 1.3; IOD 1.8; UEW 1.1; FLL 7.7; LAL 6.3; HAL 3.9; IPTL 0.6; OPTL 0.7; 3FDD 0.4; HLL 22.6; TL 7.9; FL 11.5; IMTL 0.7; 4TDD 0.4; OMTL 0.3; 1FL 0.9; 2FL 1.6; 3FL 2.8; 4FL 1.6; 1TOEL 1.5; 2TOEL 2.6; 3TOEL 3.3; 4TOEL 5.4; 5TOEL 2.5.

**Variation**: Variation in morphometric characters within the type series is shown in Table 4. In general, all paratypes agree well with the description of the holotype. Specimens vary significantly in body size, coloration of dorsal surface, form of dark “teddy-bear”-shaped brown markings on dorsum, extent of spotting on dorsum and flanks, and coloration of ventral surfaces. Males have generally much smaller body size than females: SVL in males 12.3–17.1 mm (mean 14.9±1.8 mm; *n*=7) and SVL in females 16.7–20.9 mm (mean 18.1±1.5 mm; *n*=6). Females have generally lighter coloration, with less contrast in light-brown dorsal marking and fewer bright reddish tubercles on dorsum (Figure 2B) than males (Figures 2A, 10A). In female ZMMU NAP-08274, the dorsal “teddy-bear”-like marking on the dorsum is interrupted and represented by a number of large grayish-brown irregular blotches (Figure 2B). Females have a more gray-olive tint in dorsal coloration than males (Figure 2A, B). Males have a grayish-black subgular vocal sac, whereas females have lighter grayish-white throats. Gravid females contained unpigmented eggs, visible through the semi-translucent belly skin near the groin area. Background dorsal coloration of breeding males may vary from light-gray (paratype ZMMU A5966, see Figure 10B) to beige (holotype ZMMU A5965, see Figure 10A) and light yellowish-brown (paratype ZMMU A5967, see Figure 10C).

**Figure 11:**
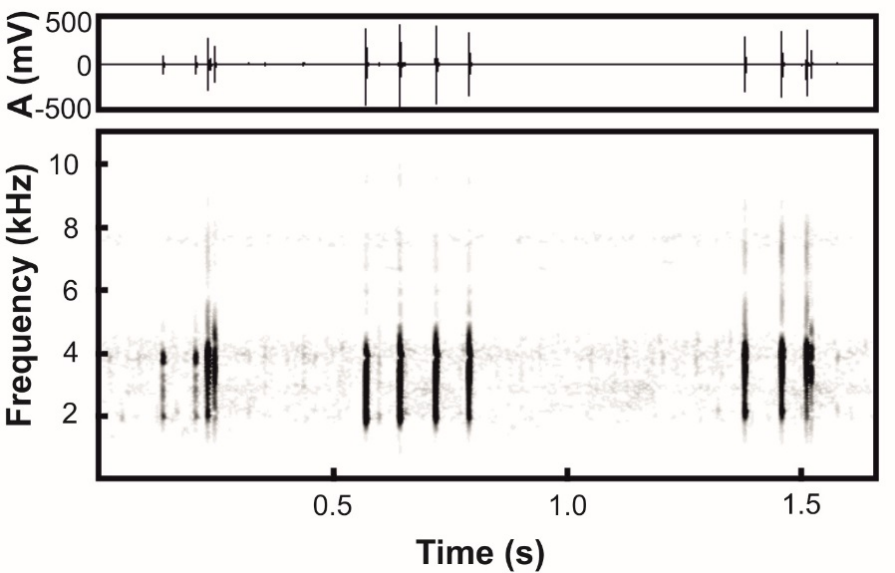
Oscillogram (top) and sonogram (bottom) of male advertisement call of *Microhyla irrawaddy* sp. nov. recorded at 21.5 °C (suburbs of Pakokku, Pakoku District, Magway Division, Myanmar)

**Advertisement call:** The *Microhyla irrawaddy* **sp. nov.** male advertisement call represents a characteristic rattling sound, resembling the sound of a ratchet to the human ear, similar to “*krrrrr… kkrrrrr… kkrrrrr…*”. Advertisement signal calls were of 0.184±0.07 s duration (0.004–0.277 s, *n*=50), consisting of 1–5 pulses (4±2, *n*=50) (Figure 11). The pulse duration was 4±0.1 ms (2–5 ms, *n*=194) and the interval between successive pulses within a call varied from 5 to 99 ms (59±2.11 ms, *n*=144). Thus, the pulse period was 63±2.12 ms (9–103 ms, *n*=144) and pulse rate varied from 11.89 to 57.14 pulses/s (17.3±1.05 pulses/s, *n*=49). The maximum amplitude frequency of pulses varied within a call from pulse to pulse and the mean value of this parameter was 3 700±570 Hz, *n*=194 (1 870–4 120 Hz). The maximum amplitude frequency of the call varied from 2 810 to 4 120 Hz (3 780±350 Hz, *n*=50).

**Natural history:** Same as *Microhyla fodiens* **sp. nov.**, the new species inhabits the dry zone of central Myanmar with a hot semi-arid savanna-like climate (see above). The new species was recorded in two habitats within the Pakokku District of Magway Division, but was always observed in close proximity to comparatively large waterbodies, including temporary and permanent pools in the Irrawaddy River Valley (Figure 7B) in suburbs of Pakokku city, or paddy fields and water reservoirs in the vicinity of Kan Pauk village.

Specimens of *Microhyla irrawaddy* **sp. nov.** were collected at night from 1830 h to 2100 h. Males did not normally sit in the open and were recorded calling from small cracks and holes in the banks of the waterbody, often hiding in footprints of buffalo hooves (Figure 10B, C). Males called from 1900 h to approximately 0200 h. Females were recorded while hiding in grass in close proximity to water-filled pools. Clutch size is unknown; one female (ZMMU A5972) laid ca. 30 eggs in a plastic bag after capture. Eggs of the new species are 0.8–0.9 mm in diameter and yellowish-white in color with a brownish animation pole. Larval stages of *Microhyla irrawaddy* **sp. nov.** are unknown. Diet of the new species is unknown.

At the type locality, *Microhyla irrawaddy* **sp. nov.** was found in sympatry with congeneric species *M*. *mukhlesuri* (Figure 2D), and also with *Fejervarya* sp.; these species shared the same breeding site with the new species. In the vicinity of Kan Pauk village, the new species was recorded in sympatry with *Microhyla fodiens* **sp. nov.**, *Kaloula pulchra*, *Fejervarya* sp., *Hoplobatrachus* cf. *tigerinus*, *Sphaerotheca* sp., and *Duttaphrynus melanostictus.*

**Distribution:** *Microhyla irrawaddy* **sp. nov.** is at present known from two closely located areas in Pakokku District of Magway Division, central Myanmar: i.e., suburbs of Pakokku city on the bank of the Irrawaddy River (the type locality) and in the vicinity of Kan Pauk village, Yesagyo Township (ca. 30 km north of type locality) (Figure 1). The species was recorded from elevations of 60 to 220 m a.s.l.. A genealogically closely related population of *Microhyla* (herein indicated as *Microhyla* sp. 2, see Table 1) was recorded from the vicinity of Chatthin in Sagaing Division of northern Myanmar by Mulcahy et al. (2018). Considering the notable genetic divergence between Sagaing and Magway populations (*P*=2.0%), further research is needed to clarify whether *Microhyla* sp. 2 is conspecific with *Microhyla irrawaddy* **sp. nov.** The actual distribution of the new species is unknown and discoveries of new localities within the middle part of the Irrawaddy River Valley are anticipated.

**Conservation status:** Currently, the actual distribution range and population trends of *Microhyla irrawaddy* **sp. nov.** remain unknown and require further study. Given the information available, we suggest *Microhyla irrawaddy* **sp. nov.** be considered as a Data Deficient species following IUCN’s Red List categories (IUCN Standards and Petitions Subcommittee, 2017).

**Etymology:** The new species name “*irrawaddy*” is given as a noun in apposition in reference to the Irrawaddy (or, officially, Ayeyarwady) River – the greatest water basin in Myanmar and western Indochina, and the cradle of Burmese civilization. The new species is known to occur in dry areas of the central part of the Irrawaddy Valley in the Magway Division, but likely has a wider distribution in the dry zone of central Myanmar. The recommended common name in English is “*Irrawaddy narrow-mouth frog*”. The recommended common name in Burmese is “*Myanmar Thaephar*”.

**Morphological comparisons:** In having toes with almost completely reduced webbing (webbing I 2–3 II 2–3 III 3–4½ IV 4½–2¾ V), *Microhyla irrawaddy* **sp. nov.** can be easily distinguished from those members of the genus that have fully developed webbing reaching to disks at most toes (usually with the exception of toe IV), including: *M. annamensis* Smith, 1923, *M. annectens*, *M. berdmorei* (including *M. fowleri* Taylor, 1934, which is considered a synonym of *M. berdmorei* by Matsui et al., 2011), *M. darevskii* Poyarkov, Vassilieva, Orlov, Galoyan, Tran, Le, Kretova & Geissler, 2014, *M. malang*, *M. mantheyi*, *M. marmorata*, *M. nanapollexa* Bain & Nguyen, 2004, *M. perparva*, *M. petrigena*, *M. pulchella* Poyarkov, Vassilieva, Orlov, Galoyan, Tran, Le, Kretova & Geissler, 2014, *M. pulverata* Bain & Nguyen, 2004, and *M. superciliaris* (detailed by Poyarkov et al., 2014).

A number of *Microhyla* species have toe webbing that reaches the level of the penultimate (distal) subarticular tubercles on at least some toes and, thus, can be easily diagnosed from *Microhyla irrawaddy* **sp. nov.**, where toe webbing is completely reduced. These species include *M. arboricola* Poyarkov, Vassilieva, Orlov, Galoyan, Tran, Le, Kretova & Geissler, 2014, *M. darreli* Garg, Suyesh, Das, Jiang, Wijayathilaka, Amarasinghe, Alhadi, Vineeth, Aravind, Senevirathne, Meegaskumbura & Biju, 2018 “2019”, *M. karunaratnei* Fernando & Siriwardhane, 1996, *M. palmipes*, *M. pulchra* (Hallowell, 1861), *M. sholigari*, and *M. zeylanica* Parker & Osman-Hill, 1948. In *M. butleri* and *M. aurantiventris* toe webbing is better developed than in *Microhyla irrawaddy* **sp. nov.** reaching the level between the two distal tubercles on the third toe.

The new species has a very slender body habitus and can be easily distinguished from those species of *Microhyla* that have a stout body habitus and enlarged spade- or shovel-shaped outer metatarsal tubercle as an adaptation for digging, including: *M. rubra*, *M. mihintalei*, *M. taraiensis*, *M. picta*, and *Microhyla fodiens* **sp. nov.** (see above). The presence of scattered red spots or dots over the dorsum was previously reported only for *M. taraiensis*; thus, with the exception of the presence of red or reddish dorsal tubercles, the new species can be distinguished from all remaining species of *Microhyla*.

The following species of *Microhyla* have notable longitudinal grooves on the dorsal surface of fingers and toes (also known as dorso-terminal grooves) and, thus, can be readily distinguished from the new species, which lacks such grooves: *M. achatina*, *M. annamensis*, *M. annectens*, *M. arboricola*, *M. aurantiventris*, *M. beilunensis* (on toes only), *M. borneensis*, *M. darreli*, *M. gadjahmadai*, *M. heymonsi* (usually present), *M. karunaratnei*, *M. kodial*, *M. malang*, *M. mantheyi*, *M. marmorata*, *M. minuta* Poyarkov, Vassilieva, Orlov, Galoyan, Tran, Le, Kretova & Geissler, 2014, *M. nanapollexa*, *M. orientalis*, *M. perparva* (on toes only), *M. petrigena*, *M. pineticola* Poyarkov, Vassilieva, Orlov, Galoyan, Tran, Le, Kretova & Geissler, 2014, *M. pulchella*, *M. pulverata*, *M. sholigari*, and *M. superciliaris* (two latter species have grooves on toes only).

*Microhyla irrawaddy* **sp. nov.** has a notably granular skin on dorsum, which readily distinguishes it from those species of *Microhyla* that have smooth or shagreened dorsal skin without prominent granular projections, including: *M. achatina*, *M. annectens*, *M. chakrapanii* Pillai, 1977, *M. darreli*, *M. fusca* Andersson, 1942, *M. gadjahmadai*, *M. heymonsi*, *M. karunaratnei*, *M. laterite*, *M. malang*, *M. marmorata*, *M. mukhlesuri*, *M. mymensinghensis*, *M. nanapollexa*, *M. perparva*, *M. pineticola*, *M. pulchella*, *M. pulverata*, *M. sholigari*, and *M. superciliaris*.

The new species has a well-developed first finger, longer than one half of the second finger length, whereas in a number of its congeners the first finger is reduced or is shorter than one half of the second finger length, including: *M. achatina*, *M. annamensis*, *M. annectens*, *M. arboricola*, *M. beilunensis*, *M. berdmorei*, *M. borneensis* (reduced to a nub), *M. darreli*, *M. fissipes*, *M. fusca*, *M. gadjahmadai*, *M. heymonsi* (smaller or equal to one half of second finger length), *M. malang*, *M. mantheyi*, *M. marmorata*, *M. mihintalei*, *M. minuta* (smaller or equal to one half of second finger length), *M. mixtura*, *M. nanapollexa* (reduced to a nub), *M. orientalis*, *M. palmipes* (reduced to a nub), *M. perparva* (reduced to a nub), *M. petrigena* (reduced to a nub), *M. picta*, *M. pineticola*, *M. pulchella*, *M. pulchra*, *M. pulverata*, and *M. superciliaris*.

*Microhyla irrawaddy* **sp. nov.** can be distinguished from *M. maculifera* Inger, 1989 from Borneo by the following characteristics: generally larger body size, adult males SVL 12.3–17.1 mm, adult females SVL 16.7–20.9 mm (vs. adult males SVL 12.0–13.3 mm, adult females 11.8 mm), two metatarsal tubercles on feet (vs. single metatarsal tubercle), dorsum irregularly covered with tubercles of various sizes (vs. two lateral rows of tubercles), and weak disks on fingers II–IV (vs. no disks on fingers). The new species can be distinguished from *M. nilphamariensis* from the lowlands of Nepal and northern India by the following characteristics: notably granular skin (vs. smooth or shagreened), first finger longer than one half of second finger length (vs. equal), and reduced webbing on toes, toe webbing formula: I 2–3 II 2–3 III 3–4½ IV 4½–2¾ V (vs. comparatively better developed foot webbing, toe webbing formula: I 2–2¾ II 2–3½ III 3–4 IV 4¼–2¾ V). The new species can also be distinguished from *M. okinavensis* by the following characteristics: smaller body size, adult males SVL 12.3–17.1 mm, adult females SVL 16.7–20.9 mm (vs. adult males SVL 22.5–28.2 mm, adult females SVL 26.5–30.8 mm), notably granular skin (vs. smooth or shagreened), presence of weak disks on fingers and toes (vs. absent), comparatively shorter hindlimbs with tibiotarsal articulation of adpressed hindlimb reaching eye level (vs. reaching snout tip), and reduced webbing on feet, toe webbing formula: I 2–3 II 2–3 III 3–4½ IV 4½–2¾ V (vs. comparatively better developed foot webbing, toe webbing formula: I 1½–2 II 1½–3¼ III 2¾–4 IV 4–2½ V).

*Microhyla irrawaddy* **sp. nov.**, can be further distinguished from its sister species *M. kodial* from southern India by the following characteristics: larger and more prominent tubercles (vs. comparatively less granular skin with small flat tubercles), no olive dorsal markings, red or orange dorsal tubercles and brown “teddy-bear”-shaped marking present dorsally (vs. olive-green markings on dorsum and lacking red-colored tubercles and brown “teddy-bear”-shaped marking on dorsum), dorsolateral line of tubercles and dark stripe present (vs. dorsolateral row of tubercles and dorsolateral dark stripe absent), and dorsal notches absent on digits (vs. short dorsal notches on finger- and toe-tips).

**Acoustic comparisons:** Comparison of advertisement call parameters is based on data from the current study and from Dehling (2010), Garcia-Rutledge & Narins (2001), Heyer (1971), Kanamadi et al. (1994), Khatiwada et al. (2017a), Kuramoto & Joshy (2006), Kurniati (2013), Le et al. (2016a, b), Matsui (2011), Nguyen et al. (2019), Vineeth et al. (2018), and Wijayathilaka & Meegaskumbura (2016). Comparison of current bio-acoustic analyses with available data is presented in Table 5.

**Table 5:** Bio-acoustic comparison of *Microhylairrawaddy *sp. nov. with other members of the genus *Microhyla*

Species	Call duration (s) (Mean±*SD*)	Call duration (s) (Min–Max)	Pulses/call (Median±interquartile range)	Pulses/call (Min–Max)	Pulse rate, pulses/s (Mean±*SD*)	Fpeak, Hz (Mean±*SD*)	Fpeak, Hz (Min–Max)	Reference
*M. irrawaddy * **sp. nov.**	0.18±0.07	0.004–0.28	4±2	1–5	17.3±1.05	3780±350	2810–4120	Present study
*M. kodial*	0.33±0.07 0.26±0.03	0.11–0.42 0.23–0.29	6±2 6±0.5	2–7 5–7	14.97±1.38 18±0.5	3752.16±233.06 3800	3359.2–4220.5 3800	Garg et al., 2019; Vineeth et al., 2018
*M. darreli*	0.65±0.06	0.59–0.74	68±5.5	63–78	105.6±1.2	3600	3600	Garg et al., 2019
*M. mymensinghensis*	0.47±0.01	0.45–0.48	21±1.1	19–22	43.1±2.9	3600±400	3500–3600	Garg et al., 2019
*M. nilphamariensis*	0.34±0.02	0.31–0.37	11±0.8	10–12	29.6±0.4	2300	2300	Garg et al., 2019
*M. achatina*	0.38±0.04 0.23±0.07	0.26–0.51 0.12–0.29	N/A 8±2	6–11 4–9	21.6±2.6 26.4±1.1	2976±143.1 3300±800	2718–3375 3200–3400	Garg et al., 2019; Kumiati, 2013
*M. malang*	0.17±0.04	0.10–0.24	N/A	4–8	30.3±1.4	2404±94	2250–2530	Dehling, 2010 Matsui, 2011
*M. petrigena*	0.13±0.03	0.07–0.18	12.1±3*	6–17	89±5	4430±322	3850–5050	Dehling, 2010
*M. heymonsi*	0.48 0.34±0.09	N/A 0.28–0.38	N/A N/A	11 11	23*** 25.7±1.6	N/A 2367±132	1700–3000 N/A	Garcia–Rutledge & Narins, 2001; Heyer, 1971; Kurniati, 2013
*M. borneensis*	0.17±0.04 N/A	0.10–0.24 0.70–0.73	5.7±1.1* N/A	4–8 2–9	30.3±1.4 N/A	2404±94 N/A	2250–2530 3000–5500	Dehling, 2010 Kurniati, 2013
*M. taraiensis*	0.75±0.12	0.70–0.91	N/A	13–14	17.3	3305.50±95.46	3433–3101	Khatiwada et al., 2017
*M. orientalis*	0.07±0.01 0.06±0.01	0.01–0.08 0.04–0.07	N/A 5±0.9	3–5 3–5	N/A 57.7±8.5	3400±100 3700	3200–3600 3700	Garg et al., 2019; Matsui et al., 2013
*M. ornata*	0.27±0.02 0.30±0.06 0.28±0.03 0.33±0.02	N/A 0.21–0.80 0.21–0.35 0.32–0.38	10.9±0.86** 13±1 11±1.75 13±0.4	N/A 9–14 9–14 13–14	35.5±1.1 41.6±1.5 38.63±1.74 37.3±1.4	2600 3100±400 2656.34±103.46 2600	N/A 2200–3400 2497.9–2842.4 2600	Garg et al., 2019; Kuramoto & Joshy, 2006; Vineeth et al., 2018; Wijayathilaka & Meegaskumbura, 2016
*M. mukhlesuri* (as *M. ornata*)	N/A	0.23–0.31	N/A	10–18	53–60***	N/A	1200–3500	Heyer, 1971
*M. sholigari*	0.73±0.04 0.93±0.05	0.66–0.82 0.90–1.01	63±4 72±3.7	56–69 70–79	86±3.84 77.3±0.4	3673.55±159.54 3400	3316.1–3962.1 3400	Garg et al., 2019; Vineeth et al., 2018
*M. laterite*	0.70±0.05 0.81±0.06	0.6–0.85 0.73–0.89	90±6.75 104±7	79–103 94–113	128.24±3.98 128.0±1.2	3670.96±97.10 3600±400	3531.4–3789.8 3500–3600	Garg et al., 2019; Vineeth et al., 2018
*M. rubra*	0.17±0.03* 0.13±0.01	0.14–0.23 0.12–0.14	18±0.2* 11±1	15–21 10–12	N/A 80.4±3.8	2268±43* 2200±700	N/A 2000–2200	Garg et al., 2019; Kanamadi et al., 1994
*M. karunaratnei*	0.87±0.1 0.87±0.13	0.7–1.17 0.77–1.00	66.5±14, 2 60±13	50–95 56–86	76.9±5.8 76.1±4.4	3300±100 3200±140	3100–3400 3100–3400	Garg et al., 2019; Wijayathilaka & Meegaskumbura, 2016
*M. zeylanica*	1.85±0.12 1.76±0.04	1.5–2 1.71–1.80	84±5 86±3.6	61–92 81–90	44.5±3.2 48.4±1.0	2600±200 2700±290	2200–2900 2300–3000	Garg et al., 2019; Wijayathilaka & Meegaskumbura, 2016
*M. mihintalei*	0.19±0.02 0.16±0.004	0.14–0.25 0.16–0.17	13±2.5 12±0.4	9–15 11–12	58.6±2.8 68.4±2.1	2100±400 2300±100	1300–2600 2300–2400	Garg et al., 2019; Wijayathilaka & Meegaskumbura, 2016
*M. butleri*	0.32±0.01	0.3–0.35	36.4±1.3**	34–40	109±4.5	3000±30	2900–3000	Nguyen et al., 2019
*M. aurantiventris*	0.14±0.02	0.11–0.23	17.9±1.45**	15–26	122.7±6.45	2100±100	1800–2200	Nguyen et al., 2019
*M. marmorata*	N/A	N/A	N/A	9–13	N/A	2857±81	2756–3015	Le et al., 2016a
*M. pulchra*	0.42±0.19	0.22–0.84	N/A	38–80	N/A	2334±44	2240–2412	Le et al., 2016b
*M. berdmorei*	N/A	0.09–0.26	N/A	3–9	33–35***	N/A	1500–1800	Heyer, 1971
*M. fissipes*	0.24±0.01	0.21–0.25	15±0.5	15–16	61.8±0.4	3000±100	3000–3100	Garg et al., 2019
*M. palmipes*	0.11±0.04	0.06–0.16	11±3.2	6–13	79.8±7.6	3500±300	3400–3500	Garg et al., 2019

Note:N/A: Data not available; *: Mean±SE; **: Mean±SD; ***: Range; Min: Minium; Max: Maximum.

*Microhyla irrawaddy* **sp. nov.** has a unique combination of acoustic parameters, such as a relatively low number of pulses per call, low pulse rate, and relatively high call frequency of maximum amplitude (see Table 5). According to most acoustic parameters, *Microhyla irrawaddy* **sp. nov.** is similar to *M. kodial* from southern India: i.e., frequency of maximum amplitude (3 780±350 Hz vs. 3 752.16±233.06 Hz), number of pulses per call (4±2 vs. 6±2), and pulse rate (17.3±1.05 pulses/s vs. 14.97±1.38 pulses/s). However, the advertisement signal of *Microhyla irrawaddy* **sp. nov.** significantly differs from *M. kodial* by call duration (0.18±0.07 s vs. 0.33±0.07 s).

Call duration in *Microhyla irrawaddy* **sp. nov.** (0.18±0.07 s) is similar to that of *M. malang* (0.17±0.04 s), *M. rubra* (0.17±0.03 s), and *M. mihintalei* (0.19±0.02 s); however, other call parameters among these species are different: for example, number of pulses per call (4±2 vs. NA, 18±0.2, and 13±2.5, respectively), pulse rate (17.3±1.05 pulses/s vs. 30.3±1.4, NA, and 58.6±2.8 pulses/s, respectively), and frequency of maximum amplitude (3 780±350 Hz vs. 2 404±94, 2 268±43, and 2 100±400 Hz, respectively).

*Microhyla irrawaddy* **sp. nov.** has a similar frequency of maximum amplitude (3 780±350 Hz) to *M. sholigari* (3 673.55±159.54 Hz) and *M. laterite* (3 670.96±97.10 Hz), but differs from these two species by values of temporal parameters, such as call duration (0.18±0.07 s vs. 0.73±0.04 and 0.70±0.05 s, respectively), number of pulses per call (4±2 vs. 63±4 and 90±6.75, respectively), and pulse rate (17.3±1.05 pulses/s vs. 86±3.84 and 128.24±3.98 pulses/s, respectively).

## DISCUSSION

Myanmar remains one of the least herpetologically studied countries in Southeast Asia. However, its vast area and diversity of lowland and montane habitats with varying climatic conditions make Myanmar a promising area for discovery of yet unknown herpetofaunal diversity (Grismer et al., 2017a, 2017b; Mulcahy et al., 2018). In recent years, significant progress has been made in describing the diversity of Myanmar amphibians (e.g., Dever, 2017; Dever et al., 2012; Grismer et al., 2018b; Pawangkhanant et al., 2018; Sheridan & Stuart, 2018; Suwannapoom et al., 2016; Wilkinson et al., 2003, 2012, 2014; Wogan, 2012; Wogan et al., 2003; Zaw et al., 2019; Zug, 2015), although almost all new species discovered have been encountered in hilly or montane wet areas covered with tropical forests. In the present paper, we describe two new species of *Microhyla* from the driest and hottest part of Myanmar–the Irrawaddy (Ayeyarwady) River Valley in the central part of the country. This area has the lowest level of precipitation in Myanmar and is characterized by a semi-arid savanna-like climate (Peel et al., 2007). Previous studies have shown that the hilly regions within the Irrawaddy Basin are notable for harboring endemic species of lizards (Grismer et al., 2018a, 2018c). Our study shows that the dry plains of the central Irrawaddy River Basin also harbor an unknown endemic diversity of frogs.

Both newly described *Microhyla* species appear to be well adapted to the seasonally dry environment, especially *Microhyla fodiens* **sp. nov.**, which is notable for its stout body habitus and large shovel-like outer metatarsal tubercle used for burrowing. Several species of *Microhyla* have developed similar adaptations and inhabit arid and often sandy areas in southern India (*M. rubra*), Sri Lanka (*M. mihintalei*), northern India and Nepal (*M. taraiensis*), and south-eastern Vietnam (*M. picta*). The phylogenetic position of *M. picta* is unknown; however, our mtDNA genealogy analysis indicated that stout-bodied burrowing species belong to at least two distinct species groups of *Microhyla* and adaptations to arid environments in the genus likely evolved independently.

Our data provide an important contribution to knowledge on the *Microhyla* fauna of Myanmar. In agreement with previous studies, we confirmed the presence of *M. heymonsi* and *M. butleri* in the country; the latter species recorded for the first time in Kachin State. A recent study by Nguyen et al. (2019) demonstrated significant divergence within *M. butleri*, which consists of two main lineages–one inhabiting southern mainland China and Taiwan, China and the other found in Indochina and the Malayan Peninsula (divergence level *P*=1.7%). The Myanmar population of *M. butleri* from Kachin State belongs to the Indochinese lineage. We also confirmed significant phylogenetic structuring within *M. heymonsi*, with Myanmar populations falling into at least two distinct lineages within the species (divergence level *P*=2.3%); however, further studies are required to clarify the phylogeographic structure of the *M. heymonsi* species complex. Our study did not confirm the results of Mulcahy et al. (2018), who assigned Myanmar populations of the former “*M. ornata*” species complex to *M. fissipes*. Results of our phylogenetic analyses clearly indicate that they belong to *M. mukhlesuri*, a species originally described from eastern Bangladesh (Hasan et al., 2014), but recently shown to inhabit almost all Indochina south of the Red River basin (Yuan et al., 2016). Thus, our work raises the number of *Microhyla* species known for Myanmar to six, and the total number of species recognized within the genus to 48.

Our knowledge on *Microhyla* diversity in Myanmar is still far from complete. Mulcahy et al. (2018) reported a population of *Microhyla* sp. from the vicinity of Chatthin in Sagaing Division, indicated in the present study as *Microhyla* sp. 2. Our phylogenetic analyses recovered this population as a sister taxon of *Microhyla irrawaddy* **sp. nov.** from the Magway Division. Though these two lineages showed a moderate level of genetic divergence (*P*=2.0%), this differentiation is comparable with genetic distances between some recognized species of *Microhyla* (e.g., *M. fissipes* and *M. mukhlesuriP*=2.4%; *M. borneensis* and *M. malangP*=2.6%; see Table 2). Hence, we consider that morphological and acoustic data are needed to test whether *Microhyla* sp. 2 is conspecific with *Microhyla irrawaddy* **sp. nov.** Further sampling and research on genetic and morphological differentiation of *Microhyla* frogs in Myanmar might lead to discovery of yet unknown lineages and species.

The phylogenetic position of *Microhyla irrawaddy* **sp. nov.** is of special interest, as it is reconstructed as a sister species with respect to *M. kodial* from southern India. The latter species was recently described and found to be the only member of the mostly Southeast Asian *M. achatina* species group reported for the Indian subcontinent (Vineeth et al., 2018). Vineeth et al. (2018) argued that the possible explanation for this biogeographic pattern could be the introduction of *M. kodial* from somewhere in Southeast Asia, and noted that the type locality of this species is adjacent to the New Mangalore Port “where timber logs imported from Myanmar, Malaysia and Indonesia used to be dumped until recently” (Vineeth et al., 2018, p. 175). According to Vineeth et al. (2018), a population of *M. kodial* could have been introduced from South East Asia and established in the lowlands of southern India several decades ago. Our discovery of a closely related but still significantly divergent lineage in arid areas of central Myanmar represents a possible “stepping stone” between Southeast Asia and India and suggests that a past dispersal event from Myanmar to the Indian subcontinent should not be excluded in explaining the biogeographic origin of *M. kodial*. Amphibians, especially small frogs such as *Microhyla*, are usually sensitive to minor changes in temperature and humidity; an unintended transfer of such miniaturized frogs on ships with timber seems an unrealistic scenario. On the other hand, the establishment of a monsoon climate and the consequent aridification starting ca. 10 mya has been shown to have influenced diversification in at least four groups of Indian lizards: i.e., *Cyrtopodion*, *Cyrtodactylus*, *Ophisops*, and *Sitana* (Agarwal et al., 2014; Agarwal & Karanth, 2015; Agarwal & Ramakrishnan, 2017; Deepak & Karanth, 2018). Progressing aridification of India could favor dispersal of species adapted to drier environments from the Eurasian mainland to the Indian subcontinent (Deepak & Karanth, 2018; Solovyeva et al., 2018). Further sampling and molecular phylogenetic and biogeographic studies are required to elucidate the biogeographic history of *Microhyla* and the processes of faunal exchange between the Indian subcontinent and Eurasian landmass.

### Key to species of *Microhyla* Tschudi, 1838 from Myanmar

The following key can be used as a guide for the identification of *Microhyla* species occurring in Myanmar.

**1a)** Body habitus stout; outer metatarsal tubercle large, shovel-like; large black blotch in inguinal region ····················*Microhyla fodiens* **sp. nov.**

**1b)** Body habitus slender or stocky; outer metatarsal tubercle small or absent ····················**2**

**2a)** Webbing on toes complete except toe IV; limbs long, tibiotarsal articulation of adpressed hindlimb reaching well beyond snout; in life inguinal region and ventral surface of thighs with yellowish tint ····················*Microhyla berdmorei*

**2b)** Webbing on toes reaching distal tubercles or rudimentary; limbs short, tibiotarsal articulation of adpressed hindlimb reaching snout or shorter; in life no yellowish tint in inguinal region and ventral surface of thighs···················· **3**

**3a)** Webbing on toe III reaching level between two distal tubercles; dorsum with dark hourglass-shaped or “teddy-bear”-shaped figure edged with light border ····················*Microhyla butleri*

**3b)** Webbing on toe rudimentary; no dark figure with light edging on dorsum ····················**4**

**4a)** Finger I length subequal to one half of finger II length; body and head sides dark-brown to black, clearly separated from light-brown coloration of dorsum; light dorsomedial stripe with black spot in scapular region ····················*Microhyla heymonsi*

**4b)** Finger I length greater than one half of finger II length; body and head sides not black, dorsomedial stripe and black scapular spot absent ····················**5**

**5a)** Medium-sized species (adult males SVL 16.5–21.0 mm); disks on digits absent; tibiotarsal articulation of adpressed hindlimb reaching snout; skin on dorsum smooth; dorsum with light-brown longitudinal lines resembling wood pattern ····················*Microhyla mukhlesuri*

**5b)** Small-sized species (adult males SVL 12.3–17.1 mm); disks on digits present; tibiotarsal articulation of adpressed hindlimb reaching eye level; skin on dorsum granular with numerous reddish tubercles ····················*Microhyla irrawaddy* **sp. nov.**
